# Digital self-management programme for pain, fatigue and faecal incontinence in inflammatory bowel disease: cost-effectiveness analysis of the IBD-BOOST randomised controlled trial

**DOI:** 10.1136/bmjgast-2025-002140

**Published:** 2026-07-01

**Authors:** Chris Roukas, Laura Miller, Fionn Cléirigh Büttner, Thomas Hamborg, Vladimir Sergeevich Gordeev, Vari Wileman, Richard C G Pollok, Sonia Saxena, Rona Moss-Morris, Ailsa Hart, James O Lindsay, Christine Norton, Borislava Mihaylova

**Affiliations:** 1 Health Economics and Policy Research Unit, Wolfson Institute of Population Health, Queen Mary University of London, London, UK; 2 Unit for Social and Community Psychiatry, Centre for Psychiatry and Mental Health (CPMH), Wolfson Institute of Population Health, Queen Mary University of London, London, UK; 3 Pragmatic Clinical Trials Unit, Wolfson Institute of Population Health, Queen Mary University of London, London, UK; 4 Department of Psychology, Institute of Psychiatry, Psychology and Neuroscience, King’s College London, London, UK; 5 Department of Gastroenterology, St. George’s University of London and NHS Trust, St George’s Hospital, London, UK; 6 School of Public Health, Imperial College London, London, UK; 7 IBD Unit, St Mark’s Hospital, London, UK; 8 Centre for Immunobiology, Blizard Institute, Faculty of Medicine and Dentistry, Queen Mary University of London, London, UK; 9 Faculty of Nursing, Midwifery and Palliative Care, King’s College London, London, UK; 10 Economics of Population Health Research Centre, Nuffield Department of Population Health, University of Oxford, Oxford, UK

**Keywords:** IBD, COST-EFFECTIVENESS, QUALITY OF LIFE

## Abstract

**Objective:**

People living with inflammatory bowel disease (IBD) frequently experience abdominal pain, fatigue and faecal incontinence that persist despite optimal medical treatment. This study aimed to assess the cost-effectiveness of IBD-BOOST, a digital, interactive, facilitator-supported self-management intervention targeting these symptoms.

**Methods:**

A cost-effectiveness analysis was conducted alongside the IBD-BOOST trial, which randomised people with IBD experiencing fatigue, pain and/or faecal incontinence to the IBD-BOOST intervention (N=391) or care as usual (N=389) over 12 months. While the IBD-BOOST intervention did not significantly improve the primary trial outcome measures (UK Inflammatory Bowel Disease Questionnaire and global rating of symptom relief), trends towards benefit were observed across study outcomes. Therefore, this analysis focuses on secondary health economic outcome measures. The cost of the intervention, including its development, facilitation and delivery, was assessed. Participants reported their health service use, out-of-pocket expenses and time off work over the previous 3 months and their health-related quality of life at baseline, 6-month and 12-month follow-up. Participants’ costs (2023 UK£) and quality-adjusted life years (QALYs) over the 12 months were compared between study arms using mixed effects models.

**Results:**

The IBD-BOOST intervention resulted in additional per participant 0.016 QALYs (95% CI 0.002 to 0.030) over the 12 months in the study and cost savings of −£304.66 (−803.51 to 194.18) for healthcare and −£39.48 (−388.09 to 309.12) for out-of-pocket costs and time off work over months 4–6 and 10–12. This resulted in cost savings of −£28 633 (95% CI −51 555 to 18 764) and −£33 568 (−64 421 to 26 198) per QALY gained with IBD-BOOST from health services and societal perspectives, respectively, and high probability of cost-effectiveness.

**Conclusion:**

The IBD-BOOST intervention is highly likely to be cost-effective for the self-management of pain, fatigue and faecal incontinence in people living with IBD.

**Trial registration number:**

ISRCTN71618461.

**WHAT IS ALREADY KNOWN ON THIS TOPIC**

- People living with inflammatory bowel disease (IBD) frequently experience symptoms of abdominal pain, fatigue and faecal incontinence that persist despite optimal medical treatment. In a large randomised controlled trial, the IBD-BOOST, a supported online self-management intervention for these symptoms in IBD, did not statistically significantly improve disease-specific quality of life or global rating of symptom relief in patients with IBD with pain, fatigue or faecal incontinence though trends towards benefit were observed across study outcomes.

**WHAT THIS STUDY ADDS**

- This cost-effectiveness analysis alongside the IBD-BOOST trial reports that the IBD-BOOST intervention improved quality-adjusted life years while reducing healthcare and other costs over the 12 months follow-up in the trial. The intervention had a high probability of being cost-effective.

**HOW THIS STUDY MIGHT AFFECT RESEARCH, PRACTICE OR POLICY**

- The IBD-BOOST intervention cost-effectively improves the quality of life of people with IBD experiencing symptoms of pain, fatigue or faecal incontinence and should be considered as an add-on to their management.

## Introduction

 Population prevalence of inflammatory bowel disease (IBD) has exceeded 0.3% in many developed countries, including the UK.^1^ People with IBD experience symptoms of pain (about 60%^2^), fatigue (41%–48%^3^) and faecal incontinence (24%^4^) more frequently and with greater severity compared with the general population even during remission.

Current clinical management of IBD focuses on controlling the intestinal inflammation and treating active IBD with immunosuppressant and biological medications. However, pain, fatigue and incontinence in people with IBD can persist despite optimal treatment, resulting in poorer quality of life^5^ and higher healthcare costs.^6^ People with IBD have expressed a preference for online self-management interventions to manage pain, fatigue and incontinence.^7^ However, such interventions remain understudied.^8^

The IBD-BOOST programme, shaped by the concerns of patients and clinicians, developed a supported online self-management intervention for these symptoms. IBD-BOOST was a pragmatic, multicentre, two-arm, parallel group superiority randomised controlled trial, with an internal pilot, of facilitator-supported online self-management versus care as usual (CAU) to relieve symptoms of pain, fatigue and faecal urgency/incontinence in people living with IBD in England, Scotland or Wales. The IBD-BOOST intervention did not improve significantly the primary outcomes, the UK Inflammatory Bowel Disease Questionnaire (IBDQ) and global rating of symptom relief (GRSR); however, an improvement in general health-related quality of life (HRQoL), reduced faecal incontinence and positive trends in other health outcomes were reported.^9^ We report the incremental cost per quality-adjusted life year (QALY) with the IBD-BOOST intervention compared with CAU from health services perspective and, separately, societal perspective over the 12-month follow-up in the IBD-BOOST trial.

## Methods

### Design, setting and participants

IBD-BOOST was a pragmatic two-arm, parallel group randomised controlled trial, of a 12-session facilitator-supported online cognitive behavioural self-management programme added to CAU versus CAU alone to manage symptoms of fatigue, pain and faecal urgency/incontinence in people with IBD. The trial design, including the integrated prospective economic evaluation, was previously reported.^10^ Adults with IBD identified by the IBD-BOOST survey^5^ as experiencing one or more symptoms of pain, fatigue or incontinence substantially impacting their quality of life (impact of 5 or more on a 0–10 scale) who could access an online intervention via a computer or mobile device were eligible. Consented participants were randomly allocated 1:1 to one of the two trial arms with stratification by (1) IBD diagnosis type (ie, Crohn’s disease (CD) versus ulcerative colitis (UC) and (2) whether they had participated in Optimise, an earlier study optimising the medical management of symptom causes.^11^ Participants assigned to the intervention group were given access to the online IBD-BOOST intervention for 6 months plus one individual telephone support session with a facilitator, plus access to online messaging with their facilitator via the IBD-BOOST platform for the first 3 months. The CAU group were informed that they would be offered access to the intervention (without a facilitator) after returning 12-month outcome measures. Baseline and follow-up assessments at 6 and 12 months post-randomisation were undertaken. This economic evaluation is reported in accordance with the Consolidated Health Economic Evaluation Reporting Standards (CHEERS) checklist.^12^

### IBD-BOOST intervention

The development and features of the IBD-BOOST intervention are described elsewhere.^13^ The intervention consisted of 12 online sessions based on a theoretically informed logic model of gut-brain psychological mechanisms that contribute to symptom maintenance. The first seven sessions were based on a cognitive behavioural model of IBD symptoms providing support on balancing activity and exercise, sleep hygiene, changing negative thoughts, coping with stress and emotions and making the most of social support. The remaining five sessions provided information on how to better manage the specific symptoms of pain, fatigue and faecal incontinence. Participants were able to access the IBD-BOOST intervention via a computer, tablet or smartphone and were encouraged to complete approximately one session per week taking about 30–60 min to complete.

During the study, participants in the intervention group were provided with facilitation to encourage use of the IBD-BOOST intervention. Each facilitator attended one-to-one and group sessions provided by a member of the research team to train them in providing the intervention. Individual and group telephone or online supervision sessions were provided to all participating facilitators to ensure the consistent and safe delivery of IBD-BOOST facilitation.

### Comparator

IBD-BOOST intervention was provided as an addition to current CAU. Participants in both groups received usual monitoring at routine or requested IBD clinic visits and/or via the local IBD helpline and care from their general practitioners.

### Health outcomes

The main health outcome measure in the health economic analysis is the QALY-derived using the EuroQol five dimension five level (EQ-5D-5L) questionnaire^14^ administered to study participants at baseline, 6-month and 12-month post-randomisation. EQ-5D-5L is a validated instrument for measuring generic HRQoL across five dimensions (mobility, self-care, usual activities, pain/discomfort and anxiety/depression), each with five levels: no problems, slight problems, moderate problems, severe problems and extreme problems. The EQ-5D-5L HRQoL utility values were calculated by mapping participant EQ-5D-5L questionnaire responses into the UK EQ-5D-3L value set using the National Institute for Health and Care Excellence (NICE) recommended mapping function.^15^ QALYs were calculated for each participant over the 12 months in the study as the area under the curve of utility values from the three time points.

### Intervention costs

Two components of costs related to the IBD-BOOST intervention were included: for intervention development and optimisation and for its web implementation, ongoing delivery and maintenance (online supplemental figure S1).

Costs for the development of the intervention, including hardware, software and work undertaken by the third-party service provider, were sourced from invoices. Costs related to activities undertaken by research staff for the intervention’s optimisation were estimated from recorded workload in the trial for each activity, the frequency of that activity and the number and grade of staff involved. The facilitator time was costed at National Health Service (NHS) grade of the role providing the service if IBD-BOOST intervention was implemented in the NHS. These included initial telephone calls and interaction with patients on the website. The costs were calculated by multiplying the time spent by the average salary for the role informed from the national reference costs.^16^ The cost of time of trial staff providing facilitator training and supervision was also included.

All intervention costs were allocated to the intervention group participants to give a per participant mean cost.

### Healthcare and other resource use and costs

Participants’ primary and secondary healthcare, and other resource use, including products and services paid by the participants and days off work due to IBD, over the previous 3 months were collected at baseline, 6 and 12 months follow-up electronically using the online self-reported IBD Resource Use Questionnaire^17^; paper questionnaires were posted on request. National reference costs were used to cost these services (online supplemental table S1). IBD medications were costed using the weighted average of the two most used generic medications according to the Prescription Cost Analysis 2022^18^ or, alternatively, the British National Formulary.^19^ Expert gastroenterologist (JOL) advised on dosage and frequency of each medication.

The analysis from the health services perspective included costs of outpatient and inpatient care, diagnostic procedures and medications. The analysis from the societal perspective also included participants’ out-of-pocket expenses on medications, products and complementary therapies, travel expenses for IBD appointments and time off work costs. Participants’ self-reported employment status and number of days off work due to IBD informed costs of time off work.

All costs were reported in 2022/2023 Great British Pounds. Where applicable, earlier unit costs were adjusted for inflation using the NHS Cost Inflation Index.^16^

### Analysis

#### Resource use

The resource use and costs at 6-month and 12-month between trial arms were compared under the missing at random assumption using separate partially nested, three-level, repeated measures, mixed-effects models that accounted for the correlation of post-randomisation outcomes within participants and the clustering of participants within facilitators (in the intervention arm only). Restricted maximum likelihood estimation was used, an unstructured covariance matrix was specified for residual errors of repeated measures, and heteroskedastic error terms (due to the partially nested design) were fitted using a Satterthwaite approximation. The following covariates, specified for the analysis of the primary outcome in IBD-BOOST, were employed: the baseline value of the respective resource use or cost outcome, IBD diagnosis type (CD or UC), whether they had participated in the Optimise study (yes or no); pain, fatigue and incontinence at baseline as continuous covariates, participant age (continuous variable) and participant gender (male or female).

#### Missing cost and QoL data

The electronic resource use and QoL questionnaires included checks ensuring data completion. We considered data missing if a participant provided a partially completed questionnaire or did not respond to a questionnaire. Individual resource use questions without responses for number of visits or contacts in the returned questionnaires were assumed to indicate that no such resource use had taken place. A small amount of missing data of total healthcare and other costs and QoL utilities at baseline were imputed using mean imputation. The remaining missing total healthcare and other costs and QoL utilities at 6 and 12 months were imputed using multiple imputation with chained equations and predictive mean matching and including a wide range of sociodemographic and clinical characteristics at baseline, including those employed in the statistical analysis of outcomes, treatment group and outcome measures. Up to 40% of study participants had at least one missing value and, therefore, 40 imputed datasets were used. To assess the plausibility of the missing at random assumption, baseline sociodemographic and clinical characteristics were compared between participants with complete and missing outcome data at 12 months.

#### Base-case economic evaluation

The primary analysis followed a pre-specified health economics analysis plan adopting the intention-to-treat principle (online supplemental material 2). A cost-utility analysis was conducted over the 12-month follow-up in IBD-BOOST. The following outcomes were compared following missing data imputation: (1) QALYs; (2) total healthcare costs and (3) other costs (out-of-pocket expenses and time off work). The comparisons employed partially nested mixed-effects models similar to those used to compare resource use with clustering effect (of patients nested within intervention facilitators) modelled in the intervention arm only. Participants in the control arm were treated as independent. In the intervention arm only, a random slope was specified to allow the effect of treatment to vary between intervention facilitators. The same covariates were employed with baseline EQ-5D utility added for QALYs outcome and baseline total healthcare and other costs for respective cost outcome.

As resource use information in the trial was collected and costed over the previous 3 months at 6-month and 12-month follow-ups, the 12-month total cost difference was estimated by doubling the estimated difference between the sums of costs over the previous 3 months at 6 and 12 months in the intervention and control groups. QALYs and costs were not discounted because the assessment period was 12 months. The NICE cost-effectiveness thresholds of £20 000–£30 000 per QALY gained were used to judge whether the intervention represents good value for money.^20^

#### Sensitivity analyses

In a sensitivity analysis, the economic analysis was performed using complete case data only. The available observations of QoL and total costs at 6-month and 12-month follow-ups were compared using similarly specified partially nested mixed-effects models, allowing estimation of mean differences in QoL and costs at each time point under the missing at random assumption. In a further sensitivity analysis, we varied the IBD-BOOST online application development plus maintenance cost from £5000 to £50 000. Finally, a sensitivity analysis with 30% higher supervision costs was performed as cost of supervision by NHS clinical staff is likely higher than research staff.

#### Subgroup analyses

Exploratory subgroup analyses were conducted to assess whether the intervention’s effects on QALYs and total costs differed in pre-specified categories of trial participants by: (1) active IBD, defined as faecal calprotectin ≥200 µg/g stool, (2) visceral sensitivity index (anxiety), (3) depression and (4) Rome IV symptom criteria for irritable bowel syndrome (IBS). Subgroup analyses were performed using the mixed effects base-case analyses but with participant category and an interaction term between the category and the intervention arm added.

All analyses were undertaken using STATA V.18.0 software. 

### Patient and public involvement

Patient and public representatives contributed to study design (including patient questionnaires) and conduct of this research and advised on study reporting.

### Role of the funding source

The funder played no role in data collection, analysis, interpretation, manuscript preparation and the decision to submit the manuscript for publication.

## Results

### Study participants

Recruitment in the IBD-BOOST trial took place between January 2020 and July 2022. Of 780 participants randomised, 391 were allocated to the IBD-BOOST intervention and 389 to CAU. The average age at randomisation was 48.6 (SD 14.4) years in the intervention group and 48.4 (SD 14.3) years in the CAU group. Most participants were of white ethnicity (95.5%), women (67.2%), with further or higher education (75.3%), employed (62.3%), never smoked (53.1%), overweight (56.4%) and living with someone (70.9%). About half of the participants were diagnosed with CD (55.4%) (table 1 and online supplemental table S2).

**Table 1:** Selected baseline demographic and clinical characteristics of IBD-BOOST trial participants

	Intervention (N=391) n (%) or mean (SD)	Control (N=389) n (%) or mean (SD)
IBD diagnosis		
Crohn’s disease	217 (55.5)	215 (55.3)
Ulcerative colitis*	174 (44.5)	174 (44.7)
Participated in Optimise study		
No	379 (96.9)	378 (97.2)
Yes	12 (3.1)	11 (2.8)
Gender		
Female	266 (68.0)	258 (66.3)
Male	124 (31.7)	129 (33.2)
Prefer not to say/to self-describe	1 (0.3)	2 (0.5)
Participant age (years)	48.6 (14.4)	48.4 (14.3)
Years since IBD diagnosis	17.2 (12.7)	17.0 (12.5)
IBD activity (faecal calprotectin test†)		
In remission	243 (82.7)	242 (79.3)
Active	51 (17.3)	63 (20.7)
PROMIS pain score‡	52.7 (10.8)	53.2 (10.6)
PROMIS fatigue score‡	57.0 (7.1)	56.9 (7.5)
PROMIS incontinence score§	7.2 (3.6)	7.4 (3.5)
VSI score¶ for anxiety	39.0 (17.2)	39.3 (16.4)
PHQ score** for depression	9.3 (5.7)	9.3 (5.7)
IBS diagnosis (using Rome IV criteria)		
No	200 (51.2)	207 (53.2)
Yes	190 (48.6)	182 (46.8)
Missing	1 (0.3)	0 (0.0)
IBDQ score††	64.8 (15.5)	63.8 (14.4)
EQ-5D utility score‡‡	0.7 (0.2)	0.7 (0.2)

* Includes ulcerative colitis and all other forms of IBD except Crohn’s disease.

† Remission (faecal calprotectin test <200 µg/g); active (faecal calprotectin test ≥200 µg/g).

‡ PROMIS pain and PROMIS fatigue T-scores: a score of 60 is 1SD higher than the mean of the reference population.

§ PROMIS bowel incontinence score: higher score indicates increased incontinence.

¶ VSI score ranges from 0 to 75, with higher scores indicating more severe anxiety.

** PHQ-9 score ranges from 0 to 27 with 0–4 indicating no or minimal depression and a score of 20–27 indicates severe depression.

†† IBDQ score ranges from 32 to 224, with higher score indicating better quality of life.

‡‡ EQ-5D utility score ranges from −0.594 to 1 where 1 is the best possible.

EQ-5D, EuroQol 5 dimension; IBD, inflammatory bowel disease; IBDQ, Inflammatory Bowel Disease Questionnaire; IBS, irritable bowel syndrome; PHQ, Patient Health Questionnaire; PROMIS, Patient-Reported Outcomes Measurement Information System; SD, standard deviation; VSI, Visceral Sensitivity Index.

The vast majority of participants completed study questionnaires online with a small number of participants requesting paper questionnaires. All 780 participants completed the study questionnaires at baseline. The 12-month post-randomisation assessment was halted early due to trial delays caused by the COVID-19 pandemic with only 613 (79%) of participants approached. There were 676 and 487 participants returning study questionnaires at 6-month and 12-month follow-ups.

The trial recruited 16 facilitators including IBD nurses (n=11), a research nurse (n=1) and non-clinical psychologists (n=4).

### Missing data

Small amount of missing baseline data (not exceeding 3.2% for any variable) and mainly limited to a small number of resource use categories was imputed in the intervention and control groups separately using mean imputation. In the intervention group, there were 313/391 (80%) participants who completed the 6-month study questionnaires and 225/391 (58%) participants who completed the 12-month questionnaires, respectively. In the control group, there were 363/389 (93%) participants who completed the 6-month questionnaires and 262/389 (67%) participants who completed the 12-month questionnaires.

### Intervention costs

Development, maintenance and delivery of the IBD-BOOST intervention together with the cost of training facilitators were estimated at £59 114 per year (online supplemental table S3) or £151 per participant allocated to the intervention.

### Resource use and costs

The patterns of other resource use and costs were similar between treatment groups and across the data collection points at baseline, 6-month and 12-month follow-up. More than a quarter of participants reported contacts with their general practitioners (GPs) in the previous 3 months (online supplemental table S4) with mean contacts with community services largest for GPs followed by pharmacists. Over one-third of the participants in both arms reported contacts with an IBD nurse and a gastroenterologist and about 20% had used an IBD advice line in the previous 3 months. Over two-thirds of participants reported having a blood test, and about a third reported having a stool sample for their IBD in the previous 3 months. More than 40% of study participants reported taking biological medications for their IBD. The mean days off work in the previous 3 months due to IBD ranged between 1.1 and 1.8 days.

**Figure 1:**
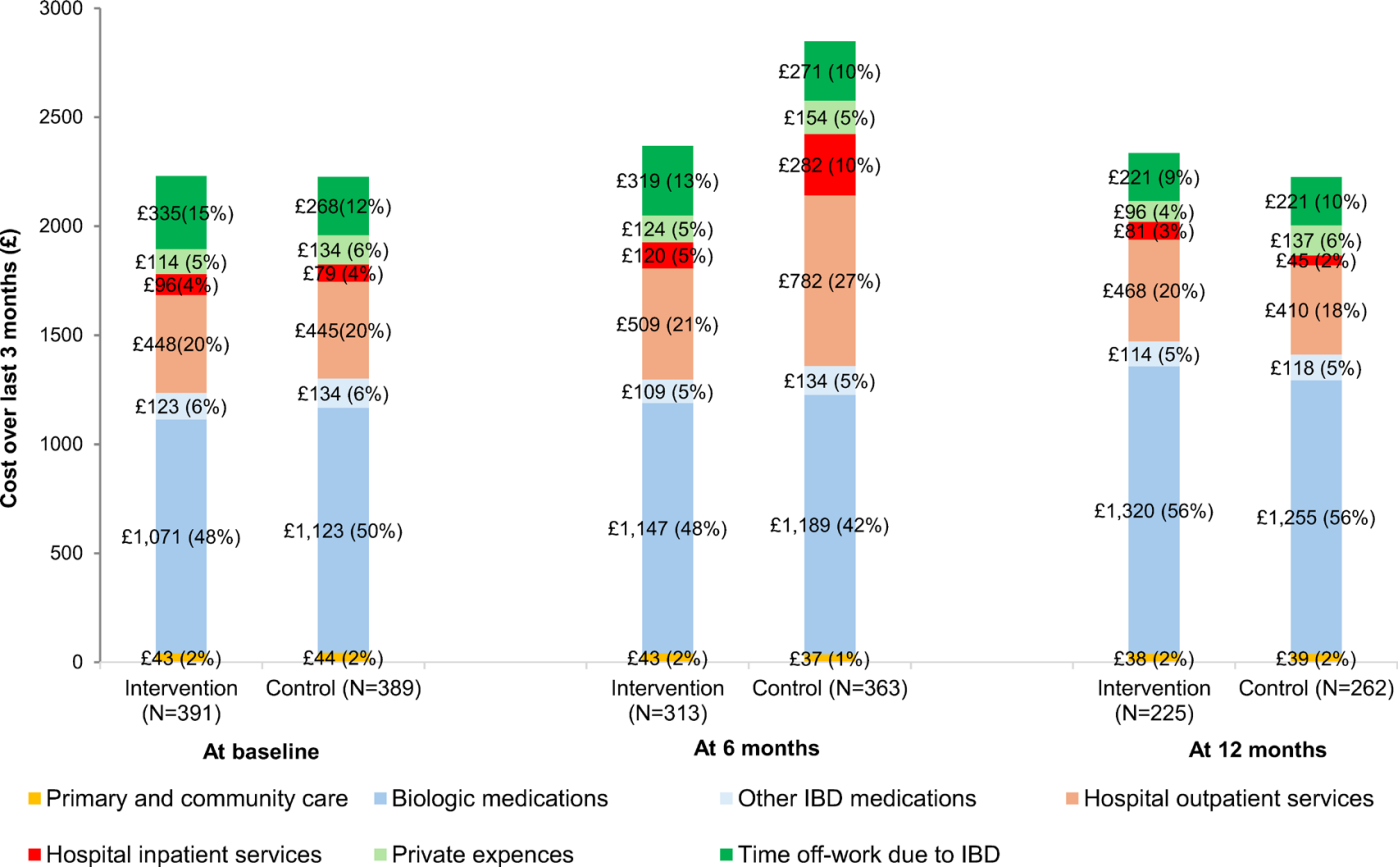
Mean costs (£) per participant in previous 3 months at baseline, 6 months and 12 months in the IBD-BOOST trial. IBD, inflammatory bowel disease.

Medication costs were by far the largest cost component with the cost of biological medication accounting for over 46% of total costs (online supplemental table S5, figure 1). The mean costs of hospital outpatient services accounted for over 17% of total costs and time off work for over 10% across the trial arms and time periods.

There were no statistically significant differences between trial arms in mean healthcare and other resources or costs in previous 3 months at 6-month and 12-month follow-ups although somewhat lower hospital costs were reported among participants allocated to the intervention at 6 months (online supplemental table S6).

### Quality-adjusted life years

The quality-of-life utility (table 2) was similar between groups at baseline (0.727 in the intervention group vs 0.728 in the control group) and slightly higher in the intervention group at the 6-month and 12-month follow-ups (0.748 vs 0.713 and 0.755 vs 0.744, respectively).

**Table 2:** Participant quality of life (EQ-5D utility scores) at baseline and at 6 and 12 months post-randomisation

	Intervention		Control	
	N (%)	Mean (SD)	N (%)	Mean (SD)
Baseline	391 (100)	0.727 (0.218)	389 (100)	0.728 (0.219)
6 months	298 (76)	0.748 (0.211)	351 (90)	0.713 (0.234)
12 months	209 (53)	0.755 (0.212)	253 (65)	0.744 (0.210)

EQ-5D, EuroQol 5 dimension; SD, standard deviation.

### Cost-effectiveness

**Figure 2:**
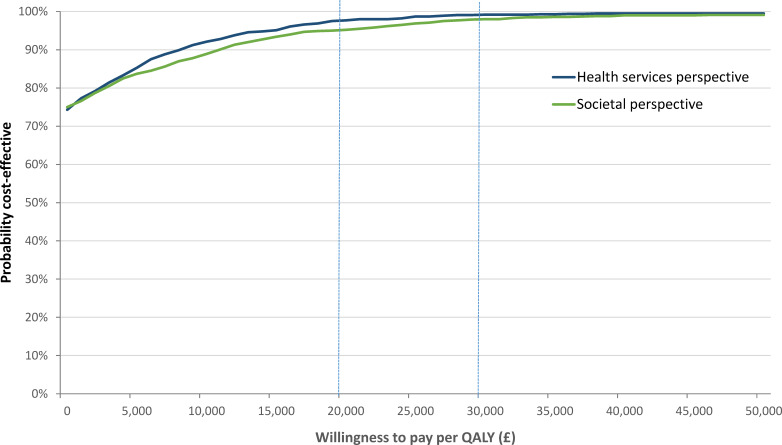
Probability the IBD-BOOST intervention is cost-effective. QALY, quality-adjusted life year.

The comparison of participants with completed QoL questionnaire and those without QoL data at 12 months indicated statistically significant differences in a number of baseline characteristics included in the missing data imputation model (online supplemental table S7); nearly the same participants completed resource use questionnaire at 12 months (data not shown). After missing data imputation and controlling for pre-specified covariates, allocation to the IBD-BOOST intervention was estimated to result in healthcare cost savings of −£304.66 (SE 254.29) over the previous 3 months at 6 and 12 months, incremental QALYs of 0.016 (SE 0.007) over the 12 months follow-up per participant and cost savings of −£28 633 per QALY gained (95% CI −51 555 to 18 764) from the health services perspective over 12 months in the study (online supplemental table S8 and table 3). Similarly, allocation to IBD-BOOST resulted in other cost savings of −£39.48 (SE 177.29) per participant over half of the 12 months follow-up and cost savings of −£33 568 per QALY gained (95% CI −64 421 to 26 198) from the wider societal perspective over 12 months in the study with the majority of the bootstrap cost-effectiveness estimates indicating increased QALYs at lower cost (online supplemental figure S2). The IBD-BOOST intervention had a probability of being cost-effective compared with CAU above 74% at willingness-to-pay threshold of £0/QALY and above 95% at £20 000/QALY or higher (figure 2).

**Table 3:** Quality of life, healthcare and other costs and cost-effectiveness of IBD-BOOST intervention: base-case analysis

Outcome	Intervention, N=391, mean (SE)	Control, N=389, mean (SE)	Mean* (95% CI), p value
Total healthcare costs over previous 3 months at 6 and 12 months (£)	3774.97 (209.66)	4142.78 (276.71)	−304.66 (−803.51 to 194.18), p=0.23
Total other costs over previous 3 months at 6 and 12 months (£)	809.76 (134.48)	833.99 (122.28)	−39.48 (−388.09 to 309.12), p=0.82
QALYs over 12 months	0.736 (0.011)	0.720 (0.011)	0.016 (0.002 to 0.030), p=0.03
Incremental cost per QALY gained (95% CI) with intervention over 12 months††			
From health services perspective		−£28 633 (−51 555 to 18 764) per QALY gained	
From societal perspective		−£33 568 (−64 421 to 26 198) per QALY gained	

* Adjusted for baseline outcome, age, gender, IBD diagnosis, treatment arm, Optimise participation, pain, fatigue and incontinence.

† Cost scaled to 12 months by doubling the reported cost difference and including intervention cost (£151.19).

CI, confidence interval; QALY, quality-adjusted life year; SE, standard error.

### Sensitivity and subgroup analyses

In the sensitivity analyses, the intervention remained cost-saving using complete cases data only (online supplemental table S9), with IBD-BOOST online application development plus maintenance cost varying from £5K to £50K (online supplemental table S10) and with 30% higher clinical supervision cost (online supplemental table S11).

In the exploratory subgroup analysis, there was no evidence of heterogeneity in IBD-BOOST’s effects on QALYs and costs between categories of participants with active disease or in remission, with or without depression, anxiety or IBS-like symptoms (table 4) with net cost savings with IBD-BOOST intervention estimated for most categories of participants.

**Table 4:** Subgroup analyses of effect of IBD-BOOST intervention in participant categories

Subgroup	Effect of intervention over 12 months of follow-up		
	Total healthcare costs over previous 3 months at 6 and 12 months, mean (SE)*	Total other costs over previous 3 months at 6 and 12 months, mean (SE)*	QALYs over 12 months, mean (SE)*
All participants (base-case analysis)	−304.66 (254.29), p=0.23	−39.48 (177.29), p=0.82	0.016 (0.007), p=0.03
IBD status (calprotectin only)	p_het_=1.0	p_het_=0.2	p_het_=0.6
In remission	−296.89 (284.43)	84.00 (187.47)	0.014 (0.008)
Active	31.29 (473.29)	66.87 (365.55)	0.009 (0.016)
PHQ-9	p_het_=0.2	p_het_=1.0	p_het_=0.8
Not depressed	−40.09 (334.57)	−39.31 (211.59)	0.017 (0.009)
Depressed	−17.00 (385.20)	276.58 (261.54)	0.014 (0.011)
VSI	−9.49 (6.05), p=0.12	−0.69 (4.35), p=0.87	0.0004 (0.0002), p=0.02
IBS-like symptoms†	p_het_=0.2	p_het_=0.5	p_het_=0.2
No	32.28 (344.54)	74.06 (211.17)	0.008 (0.010)
Yes	−105.45 (373.60)	235.86 (260.81)	0.016 (0.011)

* Adjusted for age, gender, IBD diagnosis, treatment arm, Optimise participation, pain, fatigue and incontinence. Subgroup effects estimated by adding respective subgroup factor (if not included) and subgroup by allocation to intervention interaction term.

† IBS-like symptoms as classified using ROME IV criteria for participants in remission only.

IBD, inflammatory bowel disease; IBS, irritable bowel syndrome; p_het_, test for heterogeneity between subgroups; PHQ, Patient Health Questionnaire; QALY, quality-adjusted life year; SE, standard error; VSI, Visceral Sensitivity Index.

The completed CHEERS reporting checklist is available in online supplemental table S12.

## Discussion

This is the first economic evaluation of self-management intervention for symptoms of pain, fatigue and urgency/incontinence in patients with IBD. Although the IBD-BOOST intervention did not statistically significantly improve the coprimary endpoints of disease-specific QoL or GRSR compared with CAU in the IBD-BOOST trial, trends towards benefits were observed across study outcomes.^9^ This pre-specified full economic evaluation alongside the trial reports that the IBD-BOOST intervention led to a small improvement in QALYs and possibly lower healthcare and other costs. From both health services and societal perspectives, the probability that the IBD-BOOST intervention is cost-effective exceeded 95% at willingness-to-pay threshold of £20 000 per QALY gained.

The IBD-BOOST trial found that the IBD-BOOST intervention improved the secondary outcomes of faecal incontinence at 6 and 12 months and general HRQoL at 6 months compared with CAU alone, however, while trending towards benefit, there were no differences in fatigue or pain scores. Two recent systematic reviews with meta-analyses of psychological interventions for treatment of IBD concluded that psychological interventions in adults were likely to improve quality of life but had little or no effects on disease activity.^8 21^ Two studies reporting impacts on healthcare use in the Cochrane review^8^ reported trends towards reduction in hospital days and sick-leave days,^22^ a decrease in hospital visits^23^ and a reduction in annual healthcare costs of £148 per patient.^24^ In the present study we observe lower costs across categories of healthcare and other costs among IBD-BOOST-allocated participants, though the differences between the trial arms were not statistically significant. A recent umbrella review of the efficacy of digital health technologies (DHTs) in the management of IBD reported reduced number of hospital attendances and increased treatment adherence with DHTs, supporting their role as an adjuvant to standard clinical practice in IBD.^25^

The evidence of cost-effectiveness of self-management interventions for broader chronic conditions is more promising. Economic evaluations of guided self-management interventions for people with type 2 diabetes^26^ and asthma^27^ reported the interventions to improve people’s health and reduce costs. Web-based cognitive behavioural therapies have been shown clinically effective^28^ and cost-effective^29^ for people with IBS though with more frequent contacts between the facilitators and users (2.5 hours^29^) compared with 0.5 hours in IBD-BOOST.^9^ It is of note that the adherence to intervention, defined as completing at least four sessions, was higher at 69% compared with the 57% adherence in IBD-BOOST. Future studies of self-management interventions in chronic diseases should consider the role of intensity of contacts between facilitators and users on outcomes and cost-effectiveness of interventions. Despite some evidence that guided self-management interventions are likely to be cost-effective in chronic diseases, it is difficult to generalise due to differences in populations, interventions and settings. This economic evaluation adds to the literature indicating that chronic disease self-management behavioural interventions can lead to patient benefits including better quality of life and to reduction in healthcare and other resource use^30^ and supports studying a broader set of outcomes, including general HRQoL, alongside clinical trials of self-management interventions.

The IBD-BOOST study was conducted over a 12-month time horizon and, although the intervention was delivered over the first 6 months and the difference in QoL appears to decrease between 6 and 12 months of follow-up, the 12 months horizon may not fully capture the longer-term effects of the intervention. Moreover, providing long-term access to the intervention or periodic ‘refreshers’, although incurring additional costs, may enhance long-term benefits. Future research on the longer-term effects of digital guided self-management health interventions and the role of periodic refreshers on their effectiveness and cost-effectiveness will be informative.

The IBD-BOOST study is the largest randomised controlled trial of a digital psychological intervention in IBD and the first to target the three main symptoms in a single intervention. Methodological strengths of our study include the rigorously developed intervention, robust randomised design, large sample size, good levels of participant retention and the comprehensive economic assessment of intervention’s effects on QoL and costs, including their joint uncertainties, over the 12 months in the trial. There are, however, a number of limitations to acknowledge. First, the COVID-19 pandemic led to difficulties in recruiting IBD nurses and necessitated the involvement of non-clinical psychologists and a research nurse in intervention facilitation. Moreover, the study delay due to the pandemic also required curtailing the 12-month follow-up resulting in incomplete 12-month data collection. Second, we used a self-reported questionnaire to collect the data on resource use and personal costs and, to minimise recall bias,^31^ we chose a 3 months recall period. This short recall period, however, required an assumption that mean costs over periods not covered by the questionnaire would be similar to mean costs over periods covered. Third, there was substantial amount of missing data and although both multiply imputed and complete cases analyses produced similar findings caution is advisable in view of potential differences in unmeasured characteristics. Fourth, our subgroup analyses while prespecified were not powered for and are exploratory. Therefore, these exploratory subgroup findings should not be taken to imply that the intervention is equally effective across all patient groups. Finally, the study recruited people of predominantly white ethnicity which may have been at least partially driven by the requirement to read English. Relaxing the language requirement may broaden the intervention uptake.

Our findings, in line with evidence in IBD and other chronic diseases, indicate that the IBD-BOOST-guided self-management intervention is highly likely to be cost-effective in improving general HRQoL in people with IBD experiencing symptoms of pain, fatigue or faecal incontinence and could reduce their healthcare and other costs. With the rising cost of IBD globally, the IBD-BOOST intervention could play part in improving efficiency of IBD management.

## Supplementary material

10.1136/bmjgast-2025-002140 online supplemental file 1

10.1136/bmjgast-2025-002140 online supplemental file 2

## Data Availability

Data are available upon reasonable request.
