# Supplementary material for: Digital self-management programme for pain, fatigue and faecal incontinence in inflammatory bowel disease: cost-effectiveness analysis of the IBD-BOOST randomised controlled trial
Source: BMJ Open Gastroenterol. 2026 Jul 1;13(1):e002140. doi: 10.1136/bmjgast-2025-002140 (PMC13330898; doi:10.1136/bmjgast-2025-002140)
Supplement: online supplemental file 1 [file bmjgast-13-1-s001.pdf]

**Digital self-management programme for pain, fatigue, and faecal incontinence in inflammatory bowel disease: cost-effectiveness analysis of the IBD-BOOST trial**

Supplementary material

## Contents

Figure S1: Cost components of the IBD-BOOST intervention ............ 2

Table S1: National average unit costs used in the analysis ............ 3

Table S2: Baseline characteristics of IBD-BOOST participants............ 7

Table S3: IBD-BOOST intervention development, maintenance and delivery cost............ 10

Table S4: Resource use over previous 3 months at baseline, 6 and 12 months post-randomisation, by intervention group ............ 11

Table S5: Mean costs over previous 3 months, at baseline 6 and 12 months post-randomisation (£), by intervention group ............ 14

Table S6: Effects of IBD-BOOST intervention on resource use and costs in previous 3 months at 6 and 12 months post-randomisation............ 17

Table S7 Comparison of baseline characteristics between participants with and without QoL data at 12 months follow-up ............ 18

Figure S2: Cost-effectiveness plane of the IBD-BOOST intervention ............ 21

Table S9: Quality of life, healthcare and other costs, and cost-effectiveness of IBD-BOOST intervention: a sensitivity analysis using available data only and mixed effects models ............ 22

Table S10: Sensitivity of IBD-BOOST cost-effectiveness to the annual intervention cost............ 23

Table S11: Sensitivity of IBD-BOOST cost-effectiveness to the increased supervision cost by 30% ............ 24

Table S12: CHEERS 2022 Checklist – Digital self-management programme for pain, fatigue, and faecal incontinence in inflammatory bowel disease: cost-effectiveness analysis of the IBD-BOOST trial ............ 25

Supplementary References............ 28

Figure S1: Cost components of the IBD-BOOST intervention

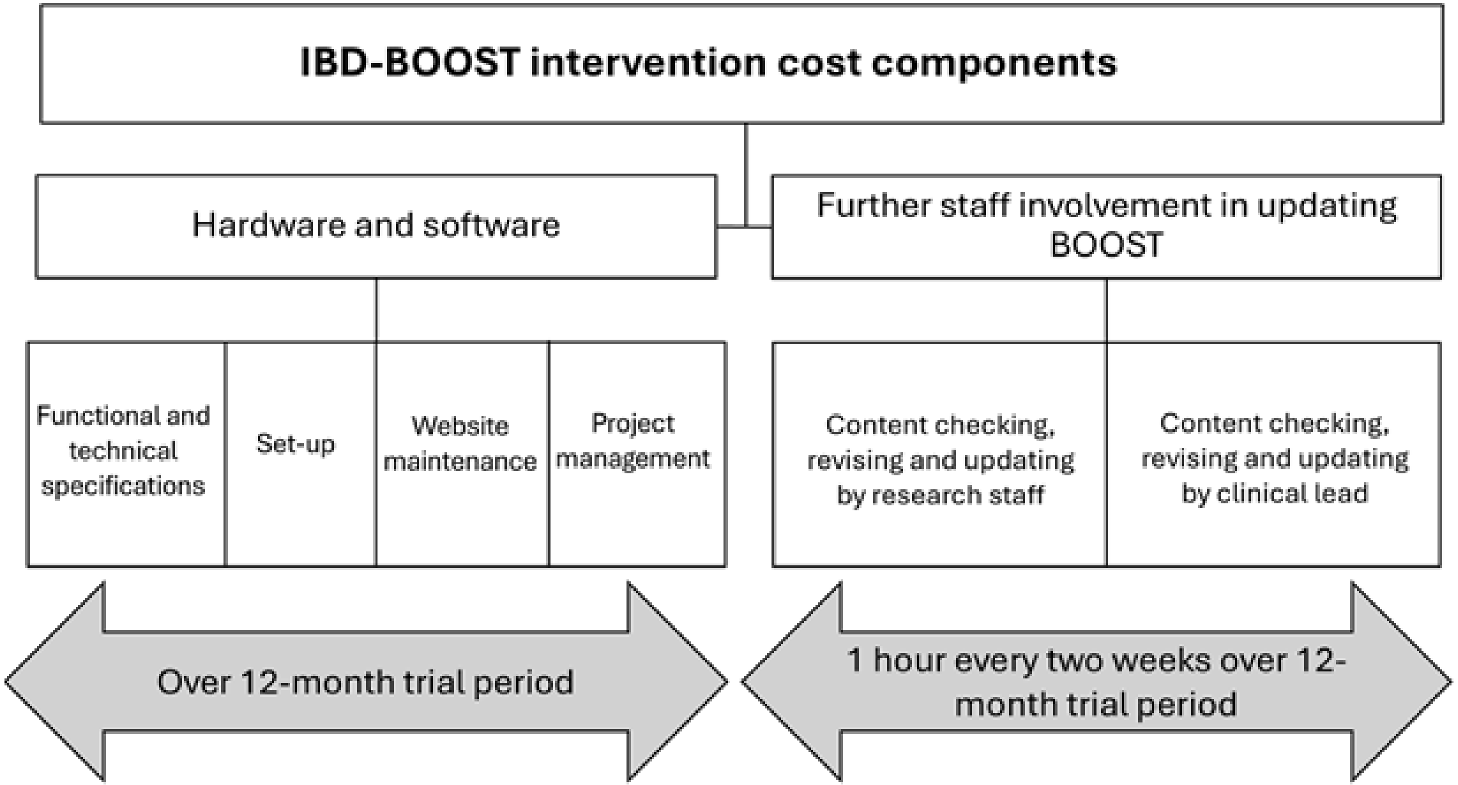

Table S1: National average unit costs used in the analysis

| Resource item | Unit cost (2022/23 UK £) | Source |
|---|---|---|
| **Community services** | | |
| General Practitioner | £52 per 10 minutes | Unit Costs of Health and Social Care 2023[2], table 9.4.2. (p. 64) |
| Practice nurse | £7.6 per 9.7 minutes | Unit Costs of Health and Social Care 2023[2], table 9.3.1. (p. 62)<br>Average consultation duration 9.7 minutes[3]. |
| Pharmacist | £9.7 per 11 minutes | Unit Costs of Health and Social Care 2023[2]: Cost per working hour Band 6 Pharmacist, table 8.2.1. (p. 56).<br>Average consultation duration 11 minutes[4]. |
| Other community services | £6.6 per 9.7 minutes | Unit Costs of Health and Social Care 2023[2]: Unit cost for Band 5 community-based scientific and professional staff, table 8.2.1. (p. 56)<br>Average consultation duration 9.7 minutes[3]. |
| **Medication** | | |
| 5ASA by mouth | £229 over 3-month period | Weighted by activity as depicted in National Summary Tables – Financial Year (Prescription Cost Analysis; England 2022/2023)[5] . |
| 5ASA as an enema or suppository | £159 over 3-month period | Weighted by activity as depicted in National Summary Tables – Financial Year (Prescription Cost Analysis; England 2022/2023)[5]. |
| Azathioprine or mercaptopurine | £8 over 3-month period | Weighted by activity as depicted in National Summary Tables – Financial Year (Prescription Cost Analysis; England 2022/2023)[5]. |
| Steroids as an enema or suppository | £470 over 3-month period | Weighted by activity as depicted in National Summary Tables – Financial Year (Prescription Cost Analysis; England 2022/2023)[5]. |
| Methotrexate by mouth or as an injection | £14 over 3-month period | Weighted by activity as depicted in National Summary Tables – Financial Year (Prescription Cost Analysis; England 2022/2023)[5]. |
| Infliximab as an infusion | £1337 over 3-month period | BNF (September 2024 - March 2025)[6] |
| Vedolizumab as an infusion | £3331 over 3-month period | BNF (September 2024 - March 2025)[6] |

| | | |
|---|---|---|
| Golimumab as an injection | £2615 over 3-month period | Weighted by activity as depicted in National Summary Tables – Financial Year (Prescription Cost Analysis; England 2022/2023)[5]. |
| Adalimumab as an injection or infusion | £2526 over 3-month period | Weighted by activity as depicted in National Summary Tables – Financial Year (Prescription Cost Analysis; England 2022/2023)[5]. |
| Ustekinumab as an injection or infusion | £3833 over 3-month period | Weighted by activity as depicted in National Summary Tables – Financial Year (Prescription Cost Analysis; England 2022/2023)[5]. |
| Steroids by mouth | £93 over 3-month period | Weighted by activity as depicted in National Summary Tables – Financial Year (Prescription Cost Analysis; England 2022/2023)[5]. |
| **Hospital services** | | |
| Inpatient (length of stay) | £355 per day | Weighted by activity as depicted in National Schedule of NHS Costs (NHS reference costs 2022/23)[7] for regular day or night admissions for IBD without interventions HRGs codes FD02E, FD02F, FD02G and FD02H. |
| Inpatient in intensive care (length of stay) | £1697 per day | Weighted by activity as depicted in National Schedule of NHS Costs (NHS reference costs 2022/23)[7] for adult critical care episodes (1 organ supported) HRG code XC06Z. |
| Surgery with intervention | £4784 per operation | Weighted by activity as depicted in National Schedule of NHS Costs (NHS reference costs 2022/23)[7] for IBD surgeries with single intervention HRGs codes FD02C and FD02D. |
| Gastroenterologist | £111 per attendance | Consultant led gastroenterology service HRG code 301 (NHS reference costs 2022/23)[7] |
| Colorectal surgeon | £144 per attendance | Consultant led colorectal surgery service code 104 (NHS reference costs 2022/23)[7] |
| Radiologist | £159 per attendance | Consultant led interventional radiology service HRG code 811 (NHS reference costs 2022/23)[7] |
| Rheumatologist | £189 per attendance | Consultant led rheumatology service HRG code 410 (NHS reference costs 2022/23)[7] |
| IBD nurse | £10.7 per 10 minutes | Unit cost for Band 6 qualified nurses, table 9.2.1. (p. 61) (Unit Costs of Health and Social Care 2023)[2] |

| | | |
|---|---|---|
| | | Average consultation duration 20 minutes[8] |
| IBD advice line | £9 per telephone call | Unit cost for a nurse-led telephone triage, table 9.6.1. (p. 67) (Unit Costs of Health and Social Care 2023)[2] |
| Stoma nurse | £10.7 per 10 minutes | Unit cost for Band 6 qualified nurses, table 9.2.1. (p. 61) (Unit Costs of Health and Social Care 2023)[2]<br>Average consultation duration 10 minutes[8] |
| A&E attendance | £245 per attendance | Weighted by activity as depicted in National Schedule of NHS Costs (NHS reference costs 2022/23)[7] for NHS emergency medicine attendances HRG codes VB03Z, VB08Z and VB09Z. |
| Dietician | £83 per attendance | Unit Costs of Health and Social Care 2023[2], table 6.1.1. (p. 36) |
| Psychologist | £320 per attendance | Clinical psychology service HRG code 656 (NHS reference costs 2022/23)[7] |
| Other specialist doctor | £169 per attendance | Weighted by activity as depicted in National Schedule of NHS Costs (NHS reference costs 2022/23)[7] for IBD-related consultant led outpatient attendances HRG codes 100, 104, 106, 160,180, 190, 191, 192, 300, 301, 656, 710, 725 and 812. |
| **Diagnostic procedures** | | |
| CT scan | £137 per test | Weighted by activity as depicted in National Schedule of NHS Costs (NHS reference costs 2022/23)[7] for Computerised Tomography Scan of One Area, 19 years and over HRG codes RD20A and RD21A. |
| MRI scan | £235 per test | Weighted by activity as depicted in National Schedule of NHS Costs (NHS reference costs 2022/23)[7] for Magnetic Resonance Imaging Scan of One Area, 19 years and over HRG codes RD01A and RD02A. |
| Colonoscopy | £889 per test | Weighted by activity as depicted in National Schedule of NHS Costs (NHS reference costs 2022/23)[7] for diagnostic Colonoscopy with Biopsy, 19 years and over HRG codes FE31Z and FE32Z. |
| Upper GI Endoscopy | £1118 per test | Weighted by activity as depicted in National Schedule of NHS Costs (NHS reference costs 2022/23)[7] for Diagnostic, Upper Gastrointestinal Tract Endoscopic Procedure with |

| | | |
|---|---|---|
| | | Colonoscopy, 19 years and over HRGs codes FE41Z and FE42Z. |
| Ultrasound | £124 per test | Weighted by activity as depicted in National Schedule of NHS Costs (NHS reference costs 2022/23)[7] for Ultrasound Scan with duration of 20 minutes and over HRG codes RD42Z and FD43Z. |
| Stool test | £28 per test | National Institute for Health and Care Excellence[9]; inflated to 2022/23 price. |
| Blood test | £10 per test | NHS laboratory charges for FBC, LFT, U&E, CRP[10]; inflated to 2022/23 price. |
| **Productivity loss** | | |
| Days off work because of ill health | £204 per day | £22.80 per hour at whole economy level in Census 2012 - Index of Labour Costs per Hour, UK: July to September 2020 (Office for National Statistics)[11]; inflated to 2022/23 price. |

5-ASA: aminosalicylates; BNF: British National Formulary; NHS: National Health Service; HRGs: Healthcare Resource Groups; A&E: Accident and Emergency; FBC: Full Blood Count; LFT: Liver Function Test; U&E: Urea and Electrolytes; CRP: C-reactive protein.

Table S2: Baseline characteristics of IBD-BOOST participants

| Characteristic | Intervention N=391 Mean (SD) or n (%) | Control N=389 Mean (SD) or n (%) |
|---|---|---|
| **Age (years)** | 48.6 (14.4) | 48.4 (14.3) |
| **Years since IBD diagnosis** | 17.2 (12.7) | 17.0 (12.5) |
| **EQ-5D index score** | 0.7 (0.2) | 0.7 (0.2) |
| **IBDQ score** | 64.8 (15.5) | 63.8 (14.4) |
| **Vaizey incontinence score** | 8.9 (5.2) | 9.2 (4.9) |
| **IBD control score** | 8.8 (4.5) | 8.9 (4.2) |
| **PHQ depression score** | 9.3 (5.7) | 9.3 (5.7) |
| **PROMIS pain score** | 52.7 (10.8) | 53.2 (10.6) |
| **PROMIS fatigue score** | 57.0 (7.1) | 56.9 (7.5) |
| **PROMIS incontinence score** | 7.2 (3.6) | 7.4 (3.5) |
| **IBD activity for CD** | 10.8 (8.0) | 11.2 (12.8) |
| **IBD activity for UC** | 1.6 (1.5) | 1.7 (1.6) |
| **VSI total score** | 39.0 (17.2) | 39.3 (16.4) |
| **IBD diagnosis** | | |
| Crohn's disease | 217 (55.5) | 215 (55.3) |
| Other IBD | 174 (44.5) | 174 (44.7) |
| **IBD activity** | | |
| In remission | 70 (17.9) | 49 (12.6) |
| Active | 224 (57.3) | 256 (65.8) |
| Missing | 97 (24.8) | 84 (21.6) |
| **Participant gender** | | |
| Female | 266 (68.0) | 258 (66.3) |
| Male | 124 (31.7) | 129 (33.2) |
| Prefer not to say | 0 (0.0) | 1 (0.3) |
| Prefer to self-describe | 1 (0.3) | 1 (0.3) |
| **Age group** | | |
| 18-25 | 20 (5.1) | 17 (4.4) |
| 26-35 | 65 (16.6) | 71 (18.3) |
| 36-45 | 77 (19.7) | 77 (19.8) |
| 46-55 | 93 (23.8) | 91 (23.4) |
| 56-65 | 74 (18.9) | 87 (22.4) |
| 66 over | 62 (15.9) | 46 (11.8) |
| **Ethnicity** | | |
| White | 371 (94.9) | 373 (95.9) |
| Mixed | 5 (1.3) | 6 (1.5) |
| Asian | 11 (2.8) | 7 (1.8) |
| Black | 1 (0.3) | 0 (0.0) |
| Other | 3 (0.8) | 2 (0.5) |
| Prefer not to say | 0 (0.0) | 1 (0.3) |
| **Education** | | |
| No formal education | 5 (1.3) | 4 (1.0) |
| School | 96 (24.6) | 85 (21.9) |

| | | |
|---|---|---|
| Further & higher education | 288 (73.7) | 299 (76.9) |
| Missing | 2 (0.5) | 1 (0.3) |
| **Work activity/Employment** | | |
| Employed | 243 (62.1) | 243 (62.5) |
| Unemployed due to Illness/disability | 31 (7.9) | 38 (9.8) |
| Other | 116 (29.7) | 108 (27.8) |
| Missing | 1 (0.3) | 0 (0.0) |
| **Living circumstances** | | |
| Living alone | 110 (28.1) | 114 (29.3) |
| Living with someone | 279 (71.4) | 274 (70.4) |
| Missing | 2 (0.5) | 1 (0.3) |
| **Participant BMI category** | | |
| Underweight | 13 (3.3) | 13 (3.3) |
| Healthy weight | 149 (38.1) | 142 (36.5) |
| Overweight | 218 (55.8) | 222 (57.1) |
| Missing | 11 (2.8) | 12 (3.1) |
| **Self-reported smoking status** | | |
| Never smoked | 209 (53.5) | 205 (52.7) |
| Ex-smoker | 158 (40.4) | 158 (40.6) |
| Current smoker | 22 (5.6) | 26 (6.7) |
| Missing | 2 (0.5) | 0 (0.0) |
| **Number of units of alcohol during average week** | | |
| None | 188 (48.1) | 188 (48.3) |
| 1-14 | 176 (45.0) | 183 (47.0) |
| 15+ | 24 (6.1) | 18 (4.6) |
| Missing | 3 (0.8) | 0 (0.0) |
| **IBD operation history status** | | |
| No | 245 (62.7) | 251 (64.5) |
| Yes | 135 (34.5) | 133 (34.2) |
| Missing | 11 (2.8) | 5 (1.3) |
| **Stoma status** | | |
| No | 369 (94.4) | 363 (93.3) |
| Yes | 22 (5.6) | 26 (6.7) |
| **Pouch status** | | |
| No | 375 (95.9) | 379 (97.4) |
| Yes | 14 (3.6) | 9 (2.3) |
| Missing | 2 (0.5) | 1 (0.3) |
| **Fistula status** | | |
| No | 335 (85.7) | 329 (84.6) |
| Yes | 17 (4.3) | 24 (6.2) |
| Unsure | 38 (9.7) | 36 (9.3) |
| Missing | 1 (0.3) | 0 (0.0) |
| **Fatigue** | | |
| No | 243 (62.1) | 248 (63.8) |
| Yes | 133 (34.0) | 130 (33.4) |
| Missing | 15 (3.8) | 11 (2.8) |
| **Pain** | | |

| | | |
|---|---|---|
| No | 281 (71.9) | 273 (70.2) |
| Yes | 105 (26.9) | 112 (28.8) |
| Missing | 5 (1.3) | 4 (1.0) |
| **Incontinence** | | |
| No | 113 (28.9) | 101 (26.0) |
| Yes | 252 (64.5) | 260 (66.8) |
| Missing | 26 (6.6) | 28 (7.2) |
| **Rome IV criterion** | | |
| No | 200 (51.2) | 207 (53.2) |
| Yes | 190 (48.6) | 182 (46.8) |
| Missing | 1 (0.3) | 0 (0.0) |
| **Depression levels** | | |
| No depression | 219 (56.0) | 221 (56.8) |
| Depressed | 172 (44.0) | 168 (43.2) |
| **Mental health conditions** | | |
| No | 272 (69.6) | 269 (69.2) |
| Yes | 119 (30.4) | 120 (30.8) |
| **Physical health conditions** | | |
| No | 223 (57.0) | 223 (57.3) |
| Yes | 168 (43.0) | 166 (42.7) |
| **Current pregnancy status** | | |
| No | 358 (91.6) | 353 (90.7) |
| Yes | 4 (1.0) | 3 (0.8) |
| Missing | 29 (7.4) | 33 (8.5) |
| **Biologic medications** | | |
| No | 325 (83.1) | 330 (84.8) |
| Yes | 45 (11.5) | 38 (9.8) |
| Missing | 21 (5.4) | 21 (5.4) |
| **Stoma or pouch** | | |
| No | 358 (91.6) | 356 (91.6) |
| Yes | 33 (8.4) | 32 (8.2) |
| Missing | 0 (0.0) | 1 (0.3) |

SD: Standard Deviation; EQ5D: EuroQoL-5 Dimension; IBDQ: Inflammatory Bowel Disease Questionnaire; IBD: Inflammatory Bowel Disease; BMI: Body Mass Index; PHQ: Patient Health Questionnaire; PROMIS: Patient-Reported Outcomes Measurement Information System; CD: Crohn’s Disease; UC: Ulcerative Colitis; VSI: Visceral Sensitivity Index

Table S3: IBD-BOOST intervention development, maintenance and delivery cost

| Activities as part of BOOST intervention | Intensity | Payment scale or source of costing | Unit cost (£) per hour | Cost (£) per year | Source |
|---|---|---|---|---|---|
| Content checking, revising and updating | 1 hour/ 2 weeks | KCL Band 5 hourly rate (£20.80) | 21 | 541 | Study data |
| Content checking, revising and updating (clinical staff) | 1 hour/ 2 weeks | KCL Band 8 hourly rate (£31.99) | 28 | 728 | Study data |
| IBD-BOOST online application development plus maintenance (external developer) | 12 months | Software developer | NA | 30,000 (one-off) | Study data |
| Facilitators' supervision | 308 hours/year | KCL Band 6 hourly rate (£21.93) | 22 | 6,769 | Study data |
| Facilitators' training (total hours & total cost of training and supervision) | 152 hours | NHS Bands 6-8 and KCL Bands 5-7 | 19 to 26 | 3,389 | Study data |
| Facilitators' training travel time and transportation to training site expenses | 23 hours | NHS Bands 6-8 and KCL Bands 5-7; Travel expenses as reported by trial facilitators | 19 to 26 | 765 | Study data |
| Delivery of BOOST: facilitators' messages | 586 hours/year | NHS Bands 6-8 and KCL Bands 5-7 | 19 to 26 | 13,017 | Study data |
| Delivery of BOOST: facilitators phone calls | 175 hours/year | NHS Bands 6-8 and KCL Bands 5-7 | 19 to 26 | 3,905 | Study data |
| **Total** | | | | **59,114** | |

KCL: King's College London (University of London) pay scale[12], NHS: National Health Service pay scale[13]

Table S4: Resource use over previous 3 months at baseline, 6 and 12 months post-randomisation, by intervention group

| | | **Baseline (N=780)** | | | | **6-month follow-up (N=676)** | | | | **12-month follow-up (N=487)** | | | |
|---|---|---|---|---|---|---|---|---|---|---|---|---|---|
| | | **Intervention (N=391)** | | **Control (N=389)** | | **Intervention (N=313)** | | **Control (N=363)** | | **Intervention (N=225)** | | **Control (N=262)** | |
| **Type of care** | **Unit** | **Users[a] n (%)** | **Contacts mean (SD)** | **Users[a] n (%)** | **Contacts mean (SD)** | **Users[a] n (%)** | **Contacts mean (SD)** | **Users[a] n (%)** | **Contacts mean (SD)** | **Users[a] n (%)** | **Contacts mean (SD)** | **Users[a] n (%)** | **Contacts mean (SD)** |
| **Primary care (NHS community-based services)** | | | | | | | | | | | | | |
| GP | Visit | 119 (30.5) | 0.7 (1.3) | 117 (30.1) | 0.7 (1.5) | 96 (31.4) | 0.7 (1.2) | 103 (29.0) | 0.6 (1.1) | 59 (27.4) | 0.6 (1.4) | 68 (26.7) | 0.6 (1.6) |
| Practice nurse | Visit | 71 (18.2) | 0.3 (0.9) | 61 (15.7) | 0.3 (1.2) | 46 (15.0) | 0.2 (0.7) | 58 (16.3) | 0.2 (0.7) | 34 (15.8) | 0.2 (0.6) | 55 (21.6) | 0.5 (1.6) |
| Pharmacist | Visit | 69 (17.7) | 0.6 (1.7) | 81 (20.9) | 0.6 (1.6) | 64 (21.0) | 0.7 (1.6) | 63 (17.8) | 0.4 (1.1) | 39 (18.1) | 0.4 (1.0) | 58 (22.8) | 0.5 (1.2) |
| Other NHS community services | Visit | 32 (8.3) | 0.1 (0.6) | 43 (11.2) | 0.2 (1.2) | 29 (9.5) | 0.1 (0.5) | 40 (11.3) | 0.2 (1.4) | 16 (7.4) | 0.1 (0.7) | 28 (11.0) | 0.1 (0.4) |
| Biological medication | Use | 158 (40.4) | N/A | 168 (43.2) | N/A | 129 (42.6) | N/A | 159 (45.0) | N/A | 101 (47.4) | N/A | 115 (45.3) | N/A |
| Other medication | Use | 241 (61.4) | N/A | 253 (65.0) | N/A | 180 (59.4) | N/A | 224 (63.3) | N/A | 132 (62.0) | N/A | 156 (61.4) | N/A |
| **Secondary care (NHS outpatient specialist appointments)** | | | | | | | | | | | | | |
| Gastroenterologist | Visit | 142 (36.3) | 0.6 (1.3) | 170 (43.7) | 0.6 (1.0) | 140 (45.8) | 0.7 (1.0) | 143 (40.3) | 0.7 (1.8) | 88 (40.9) | 0.6 (0.8) | 104 (40.8) | 0.5 (0.8) |
| Colorectal surgeon | Visit | 31 (8.0) | 0.1 (0.4) | 30 (7.7) | 0.1 (0.5) | 19 (6.2) | 0.1 (0.3) | 25 (7.0) | 0.2 (0.7) | 10 (4.7) | 0.1 (0.3) | 18 (7.1) | 0.1 (0.3) |
| Radiologist | Visit | 42 (10.8) | 0.2 (0.5) | 30 (7.7) | 0.1 (0.4) | 24 (7.8) | 0.1 (0.5) | 34 (9.6) | 0.1 (0.4) | 22 (10.2) | 0.2 (0.6) | 15 (5.9) | 0.1 (0.2) |
| Rheumatologist | Visit | 26 (6.7) | 0.1 (0.3) | 32 (8.3) | 0.1 (0.6) | 23 (7.5) | 0.1 (0.4) | 24 (6.8) | 0.1 (0.4) | 11 (5.1) | 0.1 (0.4) | 20 (7.8) | 0.1 (0.4) |

| | | | | | | | | | | | | | |
|---|---|---|---|---|---|---|---|---|---|---|---|---|---|
| IBD nurse | Visit | 181 (46.3) | 1.0 (1.6) | 191 (49.1) | 1.1 (1.8) | 118 (38.6) | 0.7 (1.2) | 152 (42.8) | 0.9 (2.0) | 87 (40.5) | 0.7 (1.2) | 87 (34.1) | 0.7 (1.6) |
| IBD advice line | Visit | 83 (21.3) | 0.6 (1.8) | 103 (26.5) | 0.7 (1.6) | 60 (19.6) | 0.4 (1.1) | 84 (23.7) | 0.6 (1.7) | 39 (18.1) | 0.4 (1.1) | 49 (19.2) | 0.4 (1.4) |
| Stoma nurse | Visit | 12 (3.1) | 0.1 (0.4) | 13 (3.3) | 0.1 (0.6) | 6 (2.0) | 0.1 (0.6) | 11 (3.1) | 0.1 (1.2) | 4 (1.9) | 0.0 (0.2) | 5 (2.0) | 0.0 (0.1) |
| A&E | Visit | 16 (4.1) | 0.0 (0.2) | 19 (4.9) | 0.1 (0.3) | 14 (4.6) | 0.1 (0.3) | 14 (3.9) | 0.0 (0.2) | 6 (2.8) | 0.1 (0.4) | 12 (4.7) | 0.1 (0.2) |
| Dietician | Visit | 33 (8.5) | 0.1 (0.5) | 23 (5.9) | 0.1 (0.7) | 19 (6.2) | 0.1 (0.4) | 24 (6.8) | 0.2 (1.2) | 17 (7.9) | 0.1 (0.4) | 13 (5.1) | 0.1 (0.3) |
| Psychologist | Visit | 21 (5.4) | 0.2 (1.3) | 18 (4.6) | 0.2 (1.2) | 13 (4.3) | 0.2 (1.2) | 20 (5.6) | 0.2 (1.3) | 7 (3.3) | 0.1 (0.9) | 10 (3.9) | 0.2 (1.1) |
| Other NHS outpatient specialist services | Visit | 22 (5.7) | 0.1 (0.6) | 22 (5.7) | 0.2 (0.8) | 16 (5.2) | 0.2 (0.8) | 22 (6.2) | 0.2 (0.7) | 11 (5.1) | 0.2 (0.9) | 16 (6.3) | 0.2 (0.6) |
| **Secondary care (NHS outpatient diagnostic appointments)** | | | | | | | | | | | | | |
| CT scan | Use | 25 (6.4) | 0.1 (0.2) | 16 (4.1) | 0.0 (0.3) | 9 (3.0) | 0.0 (0.2) | 21 (5.9) | 0.1 (0.3) | 12 (5.6) | 0.1 (0.3) | 6 (2.4) | 0.0 (0.2) |
| MRI scan | Use | 31 (8.0) | 0.1 (0.3) | 30 (7.7) | 0.1 (0.3) | 24 (7.9) | 0.1 (0.3) | 32 (9.0) | 0.1 (0.4) | 15 (7.0) | 0.1 (0.3) | 13 (5.1) | 0.1 (0.2) |
| Colonoscopy | Use | 36 (9.2) | 0.1 (0.3) | 43 (11.1) | 0.1 (0.3) | 25 (8.3) | 0.1 (0.3) | 45 (12.7) | 0.1 (0.4) | 25 (11.7) | 0.1 (0.3) | 18 (7.1) | 0.1 (0.4) |
| Endoscopy | Use | 16 (4.1) | 0.0 (0.2) | 13 (3.3) | 0.0 (0.2) | 5 (1.7) | 0.0 (0.1) | 9 (2.5) | 0.0 (0.2) | 3 (1.4) | 0.0 (0.1) | 10 (3.9) | 0.0 (0.3) |
| Ultrasound | Use | 10 (2.6) | 0.0 (0.2) | 16 (4.1) | 0.0 (0.2) | 9 (3.0) | 0.0 (0.2) | 12 (3.4) | 0.0 (0.2) | 7 (3.3) | 0.0 (0.2) | 6 (2.4) | 0.0 (0.2) |
| Stool test | Use | 141 (36.1) | 0.5 (0.8) | 142 (36.5) | 0.5 (0.9) | 104 (34.3) | 0.5 (0.7) | 123 (34.8) | 0.5 (1.1) | 74 (34.7) | 0.4 (0.7) | 76 (29.9) | 0.4 (0.6) |
| Blood test | Use | 271 (69.3) | 1.4 (2.1) | 288 (74.2) | 1.4 (1.7) | 207 (68.3) | 1.3 (1.6) | 248 (70.0) | 1.6 (2.8) | 148 (69.5) | 1.5 (2.1) | 182 (71.7) | 1.2 (1.7) |
| Other diagnostic test | Use | 28 (7.2) | 0.1 (0.5) | 34 (8.8) | 0.1 (0.4) | 18 (5.9) | 0.1 (0.4) | 24 (6.8) | 0.1 (0.3) | 14 (6.6) | 0.1 (0.3) | 13 (5.1) | 0.1 (0.2) |
| **Secondary care (NHS inpatient services)** | | | | | | | | | | | | | |

| | | | | | | | | | | | | | |
|---|---|---|---|---|---|---|---|---|---|---|---|---|---|
| Inpatient care | Length of stays (days in hospital) | 14 (3.6) | 0.2 (1.8) | 7 (1.8) | 0.2 (2.0) | 7 (2.3) | 0.2 (1.7) | 13 (3.7) | 0.4 (3.1) | 5 (2.4) | 0.2 (1.3) | 3 (1.2) | 254 (96.9) |
| **Productivity loss** | | | | | | | | | | | | | |
| Days off work owing to IBD | Days | 74 (33.6) | 1.8 (9.2) | 79 (34.2) | 1.5 (7.7) | 51 (32.3) | 1.6 (8.0) | 54 (26.9) | 1.3 (6.9) | 30 (28.0) | 1.1 (5.2) | 42 (28.6) | 1.1 (5.8) |

SD: Standard Deviation; GP: General Practitioner; NHS: National Health Service; IBD: Inflammatory Bowel Disease; A&E: Accident and Emergency; CT: Computed Tomography; MRI: Magnetic Resonance Imaging

A total of 676 (87%) participants returned the 6-month follow-up questionnaire and 487 (62%) returned the 12-months questionnaire. Item missingness ranged from 0.3%-0.8% at baseline, 2.2%-4.4% at 6 months and 2.7%-5.8% at 12 months.

[a]Number refers to the participants who answered (Y) to the question “did you use X resource use?” at each time point as percentage of all completed answers. If one category of resource use was missing, then the total number of healthcare/other care was considered missing.

Table S5: Mean costs over previous 3 months, at baseline 6 and 12 months post-randomisation (£), by intervention group

| | **Mean cost (SD) (£)** | | | | | |
|---|---|---|---|---|---|---|
| | **Baseline (N=780)** | | **6-month follow-up (N=676)** | | **12-month follow-up (N=487)** | |
| **Cost component** | **Intervention (N=391)** | **Control (N=389)** | **Intervention (N=313)** | **Control (N=363)** | **Intervention (N=225)** | **Control (N=262)** |
| **Primary care and community services** | | | | | | |
| GP | 36.1 (69.6) | 36.5 (78.8) | 34.2 (60.5) | 30.8 (59.3) | 31.7 (72.4) | 29.8 (81.1) |
| Practice nurse | 2.5 (7.1) | 2.3 (8.8) | 1.9 (5.1) | 1.9 (5.0) | 1.7 (4.6) | 3.5 (12.1) |
| Pharmacist contact | 5.4 (16.0) | 5.5 (15.6) | 6.4 (15.8) | 4.2 (10.6) | 4.1 (10.2) | 5.2 (11.3) |
| Other NHS community services | 0.4 (1.5) | 0.4 (1.5) | 0.3 (1.5) | 0.4 (1.6) | 0.3 (1.5) | 0.4 (1.6) |
| **Total primary care and community services costs** | **43.0 (78.0)** | **44.2 (85.4)** | **42.5 (68.6)** | **36.9 (66.2)** | **37.5 (76.5)** | **38.5 (88.4)** |
| Biological medication | 1070.7 (1428.7) | 1123.2 (1420.0) | 1146.5 (1480.7) | 1188.5 (1455.6) | 1319.7 (1519.2) | 1255.1 (1583.4) |
| Other medication | 123.4 (163.5) | 134.2 (183.9) | 108.9 (139.4) | 133.9 (185.7) | 114.1 (159.4) | 117.9 (168.6) |
| **Total medication costs** | **1194.2 (1425.1)** | **1257.4 (1407.5)** | **1255.4 (1474.4)** | **1322.4 (1447.0)** | **1433.8 (1526.0)** | **1372.9 (1563.5)** |
| **Hospital-based services** | | | | | | |
| **Hospital outpatient services** | | | | | | |
| Gastroenterology appointment | 65.6 (149.2) | 70.7 (109.4) | 74.7 (108.9) | 81.0 (204.9) | 61.4 (93.3) | 58.8 (89.6) |
| Colorectal surgery appointment | 14.8 (55.6) | 17.1 (67.3) | 11.3 (49.6) | 23.5 (104.0) | 8.0 (38.5) | 12.4 (49.6) |
| Radiology appointment | 26.1 (86.6) | 18.0 (69.1) | 19.7 (77.9) | 20.6 (68.7) | 28.1 (96.8) | 9.4 (37.5) |
| Rheumatology appointment | 16.1 (65.5) | 23.9 (106.6) | 17.9 (66.9) | 17.0 (77.2) | 14.9 (77.1) | 17.8 (66.8) |
| IBD nurse appointment | 10.7 (17.6) | 12.0 (19.1) | 7.4 (12.6) | 10.1 (21.7) | 8.0 (12.5) | 7.2 (17.4) |

| | | | | | | |
|---|---|---|---|---|---|---|
| IBD advice line contact | 5.1 (16.5) | 6.0 (14.3) | 4.0 (9.9) | 5.1 (15.1) | 3.7 (9.7) | 3.8 (12.3) |
| Stoma nurse appointment | 0.6 (4.5) | 0.8 (6.1) | 0.6 (6.1) | 1.3 (12.5) | 0.3 (2.3) | 0.2 (1.5) |
| A&E visit | 11.9 (60.9) | 15.1 (73.1) | 15.2 (76.6) | 11.0 (57.2) | 14.8 (92.1) | 12.5 (58.2) |
| Dietician appointment | 9.8 (40.5) | 10.7 (61.9) | 7.1 (30.7) | 13.6 (96.7) | 10.0 (37.1) | 4.9 (22.2) |
| Psychologist appointment | 77.9 (418.7) | 51.0 (373.3) | 59.6 (371.2) | 72.1 (406.8) | 44.7 (301.4) | 48.9 (339.0) |
| Other NHS outpatient specialist appointment | 14.0 (46.7) | 18.9 (53.3) | 16.0 (49.6) | 19.0 (53.5) | 12.6 (44.5) | 18.6 (52.9) |
| CT scan | 8.8 (33.6) | 6.7 (35.6) | 4.1 (23.3) | 8.9 (38.2) | 9.6 (42.0) | 3.2 (20.8) |
| MRI scan | 19.9 (69.7) | 20.5 (76.4) | 21.7 (78.2) | 25.9 (103.7) | 19.9 (79.9) | 13.0 (57.7) |
| Colonoscopy | 88.9 (302.7) | 103.1 (305.8) | 79.2 (273.5) | 125.6 (344.3) | 104.3 (286.8) | 77.0 (316.6) |
| Endoscopy | 48.7 (242.2) | 37.4 (201.2) | 18.4 (142.7) | 34.7 (242.9) | 15.7 (132.1) | 52.8 (293.5) |
| Ultrasound | 3.5 (22.4) | 5.4 (26.9) | 4.5 (27.3) | 4.6 (25.2) | 4.7 (26.5) | 2.9 (18.9) |
| Stool test | 13.6 (23.5) | 14.3 (26.0) | 12.8 (20.7) | 14.4 (30.7) | 12.5 (19.5) | 10.4 (17.7) |
| Blood test | 14.4 (20.6) | 14.0 (16.9) | 13.4 (16.2) | 16.0 (27.6) | 14.7 (21.0) | 12.5 (16.6) |
| **Total hospital outpatient costs** | **447.9 (813.0)** | **445.1 (811.4)** | **388.5 (661.1)** | **500.7 (966.5)** | **387.1 (690.4)** | **364.9 (897.3)** |
| **Hospital inpatient services** | | | | | | |
| Inpatient stay cost | 71.7 (639.4) | 54.8 (709.7) | 72.6 (598.8) | 146.4 (1105.3) | 58.3 (466.0) | 26.6 (271.1) |
| IBD operation | 24.5 (341.7) | 24.6 (342.6) | 47.4 (474.4) | 135.1 (1388.1) | 22.5 (327.8) | 18.8 (300.2) |
| **Total hospital inpatient costs** | **96.2 (780.5)** | **79.4 (829.7)** | **120.0 (979.4)** | **281.6 (2160.4)** | **80.8 (581.3)** | **45.4 (519.6)** |
| **Total hospital costs** | **545.9 (1333.0)** | **526.4 (1381.0)** | **628.6 (2258.0)** | **1063.8 (4983.8)** | **467.9 (1061.7)** | **410.3 (1121.7)** |
| **Total healthcare costs** | **1786.6 (2107.7)** | **1823.2 (2145.5)** | **1806.2 (2036.0)** | **2141.7 (3441.7)** | **1939.3 (2004.6)** | **1821.1 (2148.2)** |
| **Private costs** | | | | | | |
| Products and services | 104.3 (173.2) | 122.3 (233.8) | 112.4 (193.1) | 138.3 (307.9) | 86.7 (141.6) | 125.8 (390.5) |
| Transport | 9.7 (24.0) | 13.6 (52.7) | 10.9 (33.8) | 13.8 (42.8) | 9.2 (17.5) | 11.3 (43.2) |
| Other expenses (e.g. childminder, carer) | 0.7 (7.5) | 0.5 (5.8) | 0.4 (5.8) | 1.4 (12.4) | 0.0 (0.0) | 0.2 (2.6) |

| | | | | | | |
|---|---|---|---|---|---|---|
| **Total private expenses** | **114.1 (174.3)** | **134.3 (242.7)** | **123.7 (200.3)** | **153.5 (319.3)** | **96.0 (145.5)** | **137.4 (393.1)** |
| **Productivity costs (days off work)** | | | | | | |
| Total productivity costs | 334.5 (1675.7) | 267.8 (1395.3) | 318.6 (1626.9) | 270.7 (1417.6) | 220.8 (1050.8) | 220.7 (1188.3) |
| **Total other costs** | **447.8 (1705.6)** | **384.7 (1399.3)** | **443.5 (1681.9)** | **424.5 (1463.1)** | **317.2 (1063.9)** | **360.0 (1271.7)** |
| **Total societal costs** | **2208.0 (3119.4)** | **2224.2 (2863.8)** | **2243.6 (2839.5)** | **2569.8 (4080.1)** | **2265.6 (2555.7)** | **2156.6 (2711.2)** |

SD: Standard Deviation; GP: General Practitioner; NHS: National Health Service; IBD: Inflamatory Bowel Disease; A&E: Accident and Emergency; CT: Computed Tomography; MRI: Magnetic Resonance Imaging

A total of 676 (87%) participants returned the 6-month follow-up questionnaire and 487 (62%) returned the 12-months questionnaire. Item missingness ranged from 0.3%-0.8% at baseline, 2.2%-4.4% at 6 months and 2.7%-5.8% at 12 months.

Table S6: Effects of IBD-BOOST intervention on resource use and costs in previous 3 months at 6 and 12 months post-randomisation

| Outcome | Number participant observations at 6 and 12 months | Mean difference (Intervention - Control) (SE), p-value* At 6 months | At 12 months |
|---|---|---|---|
| **Resource use over previous 3 months (per person)** | | | |
| Primary care visits, number visits | 1131 | 0.04 (0.19), 0.849 | -0.21 (0.24), 0.377 |
| Biologic medication use, number people | 1124 | -0.01 (0.03), 0.658 | 0.01 (0.03), 0.993 |
| Other medication use, number people | 1124 | 0.01 (0.02), 0.714 | 0.05 (0.03), 0.045 |
| Hospital outpatient visits, number visits | 1130 | -0.47 (0.78), 0.544 | 0.12 (0.76), 0.878 |
| Hospital diagnostic tests, number tests | 1124 | -0.38 (0.24), 0.108 | 0.30 (0.23), 0.190 |
| Hospital inpatient nights, number | 1124 | -0.23 (0.19), 0.225 | 0.05 (0.10), 0.590 |
| Days off work, number | 1110 | 0.24 (0.59), 0.684 | -0.17 (0.47), 0.721 |
| **Costs over previous 3 months (£, per person)** | | | |
| Primary care | 1131 | 7.13 (4.97), 0.151 | -0.27 (7.20), 0.970 |
| Biologic medication | 1124 | -16.60 (64.25), 0.796 | 7.23 (92.71), 0.938 |
| Other medication | 1124 | -9.42 (9.48), 0.320 | 8.42 (11.29), 0.456 |
| Hospital outpatient | 1124 | -277.58 (173.55), 0.110 | 18.17 (106.70), 0.865 |
| Hospital inpatient | 1124 | -168.24 (124.55), 0.177 | 22.09 (51.61), 0.669 |
| Out-of-pocket expenses | 1122 | -2.89 (16.44), 0.860 | -24.14 (22.81), 0.290 |
| Productivity loss | 1110 | 49.25 (120.89), 0.684 | -33.90 (95.03), 0.721 |
| **Total healthcare costs** | 1124 | **-312.55 (169.50), 0.065** | **-22.31 (136.07), 0.870** |
| **Total other costs** | 1108 | **28.60 (124.11), 0.818** | **-86.46 (99.16), 0.383** |

* All available observations included in the analysis. Missing values at baseline (<1%) were imputed using the mean of observed values for respective resource use/cost at baseline. Separate mixed effects models for each reported outcome and total healthcare and other costs including adjustments for respective resource use/cost at baseline, age, gender, IBD diagnosis, treatment arm, Optimize participation, pain, fatigue and incontinence; SE: standard error.

Table S7 Comparison of baseline characteristics between participants with and without QoL data at 12 months follow-up

| | Non-missing QoL (N=462) n (%) or mean (SD) | | Missing QoL (N=318) n (%) or mean (SD) | | p-value |
|---|---|---|---|---|---|
| **IBD diagnosis [N(%)]** | | | | | |
| Crohn's disease | 269 | (58.2%) | 163 | (51.3%) | 0.054 |
| Ulcerative colitis[1] | 193 | (41.8%) | 155 | (48.7%) | |
| **Gender** | | | | | |
| Female | 293 | (63.4%) | 231 | (72.6%) | 0.014 |
| Male | 168 | (36.4%) | 85 | (26.7%) | |
| Prefer not to say/to self-describe | 1 | (0.2%) | 2 | (0.6%) | |
| **Age (years)** | 49.6 | (14.1) | 46.9 | (14.6) | 0.009 |
| **BMI category** | | | | | |
| Underweight | 182 | (39.4%) | 109 | (34.3%) | 0.175 |
| Healthy weight | 14 | (3.0%) | 12 | (3.8%) | |
| Overweight | 249 | (53.9%) | 191 | (60.1%) | |
| Missing | 17 | (3.7%) | 6 | (1.9%) | |
| **Ethnicity** | | | | | |
| White | 438 | (94.8%) | 306 | (96.2%) | 0.353 |
| Other | 24 | (5.2%) | 12 | (3.8%) | |
| **Educational level** | | | | | |
| No formal education | 4 | (0.9%) | 5 | (1.6%) | 0.512 |
| Secondary school | 66 | (14.3%) | 60 | (18.9%) | |
| Sixth form | 32 | (6.9%) | 23 | (7.2%) | |
| Further education | 124 | (26.8%) | 83 | (26.1%) | |
| Higher education | 234 | (50.6%) | 146 | (45.9%) | |
| Missing | 2 | (0.4%) | 1 | (0.3%) | |
| **Employment** | | | | | |
| Employed | 267 | (57.8%) | 219 | (68.9%) | 0.005 |
| Unemployed due to IBD | 40 | (8.7%) | 29 | (9.1%) | |
| Other (i.e. student or retired) | 154 | (33.3%) | 70 | (22.0%) | |
| Missing | 1 | (0.2%) | 0 | (0.0%) | |
| **PROMIS pain score[2]** | 52.0 | (10.5) | 54.4 | (10.6) | 0.002 |
| **PROMIS fatigue score[3]** | 56.2 | (7.2) | 58.2 | (7.0) | <0.001 |
| **PROMIS incontinence score[3]** | 7.0 | (3.2) | 7.7 | (3.7) | 0.005 |
| **VSI score[4] for anxiety** | 36.9 | (16.9) | 42.4 | (16.2) | <0.001 |
| **PHQ score[5] for depression** | 8.4 | (5.4) | 10.6 | (5.8) | <0.001 |
| **IBS diagnosis (using Rome IV criteria)** | | | | | |
| No | 263 | (56.9%) | 144 | (45.3%) | 0.003 |
| Yes | 199 | (43.1%) | 173 | (54.4%) | |
| Missing | 0 | (0.0%) | 1 | (0.3%) | |

| | | | | | |
|---|---|---|---|---|---|
| **IBDQ score[6]** | 61.8 | (14.2) | 67.9 | (15.3) | <0.001 |
| **EQ-5D utility score[7]** | 0.75 | (0.20) | 0.70 | (0.24) | <0.001 |

p-values based on univariate comparisons with continuous variables compared using t test and categorical variables compared using Pearson's chi-square test.

BMI: Body Mass Index; EQ-5D: EuroQol 5 Dimension; IBD: Inflammatory Bowel Disease; IBDQ: Inflammatory Bowel Disease Questionnaire; IBS: Irritable Bowel Syndrome; PHQ: Patient Health Questionnaire; PROMIS: Patient-Reported Outcomes Measurement Information System; SD: Standard Deviation; VSI: Visceral Sensitivity Index

[1]includes ulcerative colitis and all other forms of IBD except Crohn's disease

[2]PROMIS pain and PROMIS fatigue T-scores: a score of 60 is one SD higher than the mean of the reference population.

[3]PROMIS bowel incontinence score: higher score indicates increased incontinence.

[4]VSI score ranges from 0 to 75, with higher scores indicating more severe anxiety.

[5]PHQ-9 score ranges from 0-27 with 0–4 indicating no or minimal depression and a score of 20–27 indicates severe depression.

[6]IBDQ score ranges from 32 to 224, with higher score indicating better quality of life.

[7]EQ5D utility score ranges from -0.594 to 1 where 1 is the best possible.

Supplementary Table S8: Participant quality of life (EQ-5D utility scores) at 6 and 12 months post-randomisation following multiple imputation of missing data

| | **Intervention (N=391)** **Mean (SE)** | **Control (N=389)** **Mean (SE)** | **p-value**[1] |
|---|---|---|---|
| QoL at baseline | 0.727 (0.011) | 0.728 (0.011) | |
| QoL at 6 months | 0.742 (0.112) | 0.713 (0.012) | 0.01 |
| QoL at 12 months | 0.734 (0.013) | 0.725 (0.012) | 0.56 |
| QALYs over 12 months | 0.736 (0.011) | 0.720 (0.011) | 0.03 |

SE: standard error

[1]adjusted for baseline QoL, age, gender, IBD diagnosis, treatment arm, Optimize participation, pain, fatigue and incontinence

Figure S2: Cost-effectiveness plane of the IBD-BOOST intervention

a) From health services perspective

b) From societal perspective

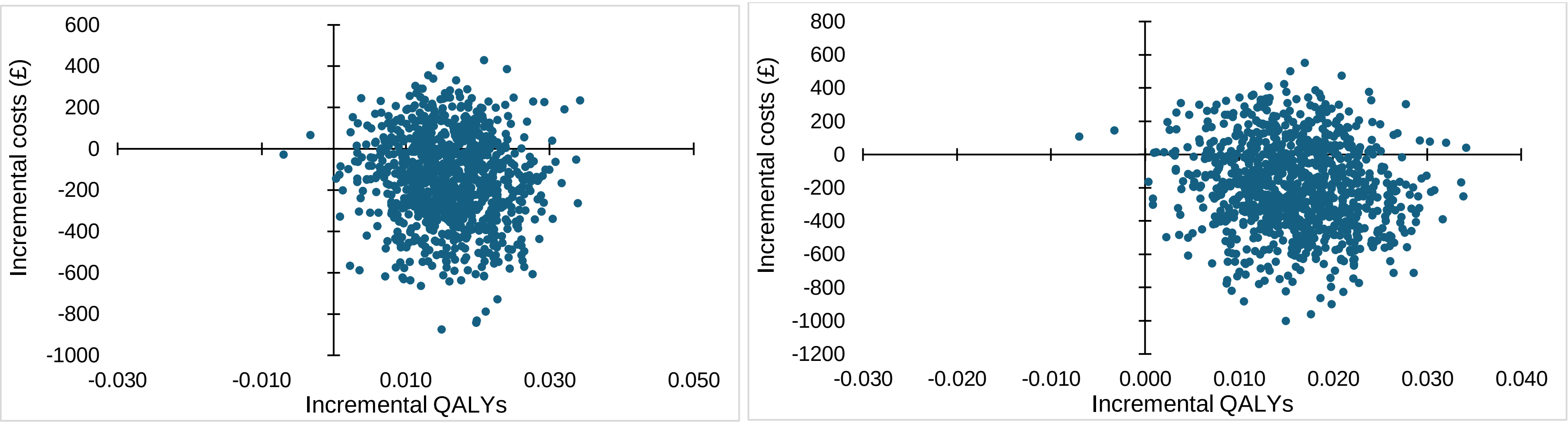


QALY: Quality-Adjusted Life Year

Table S9: Quality of life, healthcare and other costs, and cost-effectiveness of IBD-BOOST intervention: a sensitivity analysis using available data only and mixed effects models

| **Outcome** | **Intervention effect at 6 months*** **Mean (SE), p-value** | **Intervention effect at 12 months*** **Mean (SE), p-value** |
|---|---|---|
| Total healthcare costs over previous 3 months (£)[†] | -312.55 (169.50), 0.065 | -22.31 (136.07), 0.870 |
| Total other costs over previous 3 months (£)[†] | 28.60 (124.11), 0.818 | -86.46 (99.16), 0.383 |
| QoL utility | 0.028 (0.0109), 0.010 | 0.009 (0.0135), 0.490 |
| | **Intervention effects over 12 months follow-up** **Mean (95% CI)** | |
| Incremental QALYs over 12 months[§] | 0.016 (0.008; 0.030) | |
| Incremental total healthcare costs over previous 3 months at 6 and 12 months | -669.72 (-722.78; 135.86) | |
| Incremental other costs over previous 3 months at 6 and 12 months | -115.72 (-361.07, 268.90) | |
| **Incremental cost per QALY gained over 12 months[‡]** | -£31,910 | |
| • From health services perspective | -£39,031 | |
| • From societal perspective | | |

Note: Number of observations contributing to the mixed effects model: total healthcare costs (n=1,124), total other costs (n=1,108), QoL utility (n=1,111).

*adjusted for age, gender, IBD diagnosis, treatment arm, Optimize participation, pain, fatigue and incontinence; †over previous 3 months; §estimated as the sum of QALYs in 0 to 6 and 6 to 12 months assuming all participants were alive over the 12 months post randomisation; ‡ cost scaled to 12 months by doubling the reported cost differences and including intervention cost (£151.19 per participant); SE: standard error; 95% CI: 95% confidence interval

Table S10: Sensitivity of IBD-BOOST cost-effectiveness to the annual intervention cost

| Annual cost of online intervention and ongoing maintenance | Incremental cost (£) per QALY gained (95% CI) (health services perspective) | Incremental cost (£) per QALY gained (95% CI) (societal perspective) |
|---|---|---|
| £5,000 | -32,630 (-64,503, 12,469) | -37,565 (-71,196, 20,584) |
| £10,000 | -31,830 (-61,916, 13,906) | -36,765 (-68,636, 21,689) |
| £15,000 | -31,031 (-60,072, 15,153) | -35,966 (-67,562, 23,026) |
| £20,000 | -30,232 (-56,861, 16,305) | -35,167 (-66,487, 24,116) |
| £25,000 | -29,433 (-53,897, 17,483) | -34,368 (-65,496, 25,167) |
| £30,000 (base-case analysis) | -28,633 (-51,555, 18,764) | -33,568 (-64,421, 26,198) |
| £35,000 | -27,834 (-50,151, 19,972) | -32,769 (-63,196, 27,230) |
| £40,000 | -27,035 (-48,748, 21,107) | -31,970 (-61,789, 29,461) |
| £45,000 | -26,236 (-47,344, 22,372) | -31,171 (-60,381, 30,852) |
| £50,000 | -25,436 (-45,218, 23,183) | -30,371 (-60,207, 32,008) |

CI: confidence interval; QALY: quality-adjusted life year

Table S11: Sensitivity of IBD-BOOST cost-effectiveness to the increased supervision cost by 30%

| Activities as part of BOOST intervention | Intensity | Payment scale or source of costing | Unit cost (£) per hour | Cost (£) per year | Source |
|---|---|---|---|---|---|
| Facilitators’ supervision | 308 hours/year | Increased supervision cost by 30% compared to baseline analysis (£28.51) | 29 | 8,832 | Assumption for cost of clinical staff time |
| **Total** | | | | **61,177** | |
| **Incremental cost per QALY gained over 12 months‡ (95% CI)** | | | | | |
| • From health services perspective | -£ 28,304 (-50,965, 19,232) | | | | |
| • From societal perspective | -£ 33,239 (-63,970, 26,632) | | | | |

Note: assumed that all other IBD-BOOST intervention development, maintenance and delivery cost components remained unchanged compared to the baseline analyses (as costed in Table S3)

KCL: King’s College London (University of London) pay scale[12], NHS: National Health Service pay scale[13]; 95% CI: 95% confidence interval

‡ cost scaled to 12 months by doubling the reported cost differences and including intervention cost (£156.46 per participant).

Table S12: CHEERS 2022 Checklist – Digital self-management programme for pain, fatigue, and faecal incontinence in inflammatory bowel disease: cost-effectiveness analysis of the IBD-BOOST trial

| | | | |
|---|---|---|---|
| **Title** | | | |
| | 1 | Identify the study as an economic evaluation and specify the interventions being compared. | Start of paper |
| **Abstract** | | | |
| | 2 | Provide a structured summary that highlights context, key methods, results, and alternative analyses. | Start of paper, Abstract |
| **Introduction** | | | |
| **Background and objectives** | 3 | Give the context for the study, the study question, and its practical relevance for decision making in policy or practice. | Introduction |
| **Methods** | | | |
| **Health economic analysis plan** | 4 | Indicate whether a health economic analysis plan was developed and where available. | Available as supplementary material 2 |
| **Study population** | 5 | Describe characteristics of the study population (such as age range, demographics, socioeconomic, or clinical characteristics). | Methods-Design, setting and participants, Table 1 Supplementary material pg. 7 Table S2 |
| **Setting and location** | 6 | Provide relevant contextual information that may influence findings. | Methods-Design, setting and participants |
| **Comparators** | 7 | Describe the interventions or strategies being compared and why chosen. | Methods-IBD-BOOST intervention; Comparator |
| **Perspective** | 8 | State the perspective(s) adopted by the study and why chosen. | Methods |
| **Time horizon** | 9 | State the time horizon for the study and why appropriate. | Methods- Base-case economic evaluation |
| **Discount rate** | 10 | Report the discount rate(s) and reason chosen. | Methods - Base-case economic evaluation |
| **Selection of outcomes** | 11 | Describe what outcomes were used as the measure(s) of benefit(s) and harm(s). | Methods-Health Outcomes |
| **Measurement of outcomes** | 12 | Describe how outcomes used to capture benefit(s) and harm(s) were measured. | Methods-Health Outcomes |
| **Valuation of outcomes** | 13 | Describe the population and methods used to measure and value outcomes. | Methods-Health Outcomes |
| **Measurement and valuation of resources and costs** | 14 | Describe how costs were valued. | Methods-Costs of the intervention; Healthcare and other resource use and costs |

| | | | |
|---|---|---|---|
| **Currency, price date, and conversion** | 15 | Report the dates of the estimated resource quantities and unit costs, plus the currency and year of conversion. | Methods - Healthcare and other resource use and costs |
| **Rationale and description of model** | 16 | If modelling is used, describe in detail and why used. Report if the model is publicly available and where it can be accessed. | Not applicable |
| **Analytics and assumptions** | 17 | Describe any methods for analysing or statistically transforming data, any extrapolation methods, and approaches for validating any model used. | Methods - Analysis |
| **Characterising heterogeneity** | 18 | Describe any methods used for estimating how the results of the study vary for subgroups. | Methods- Subgroup analyses |
| **Characterising distributional effects** | 19 | Describe how impacts are distributed across different individuals or adjustments made to reflect priority populations. | Methods- Subgroup analyses |
| **Characterising uncertainty** | 20 | Describe methods to characterise any sources of uncertainty in the analysis. | Methods- Analysis/Base-case economic evaluation |
| **Approach to engagement with patients and others affected by the study** | 21 | Describe any approaches to engage patients or service recipients, the general public, communities, or stakeholders (such as clinicians or payers) in the design of the study. | Methods-Patient and Public involvement |
| **Results** | | | |
| **Study parameters** | 22 | Report all analytic inputs (such as values, ranges, references) including uncertainty or distributional assumptions. | Results, Supplementary material Figure S1, Table S1 |
| **Summary of main results** | 23 | Report the mean values for the main categories of costs and outcomes of interest and summarise them in the most appropriate overall measure. | Results<br>Tables 2-4, Figure 1, Supplementary Tables S3-S5 |
| **Effect of uncertainty** | 24 | Describe how uncertainty about analytic judgments, inputs, or projections affect findings. Report the effect of choice of discount rate and time horizon, if applicable. | Figure 2 pg. 34; Supplementary Tables S6- S11; Supplementary figure S2 |
| **Effect of engagement with patients and others affected by the study** | 25 | Report on any difference patient/service recipient, general public, community, or stakeholder involvement made to the approach or findings of the study | Methods - Patient and public involvement |
| **Discussion** | | | |
| **Study findings, limitations, generalisability, and current knowledge** | 26 | Report key findings, limitations, ethical or equity considerations not captured, and how these could affect patients, policy, or practice. | Discussion |
| **Other relevant information** | | | |

| | | | |
|---|---|---|---|
| **Source of funding** | 27 | Describe how the study was funded and any role of the funder in the identification, design, conduct, and reporting of the analysis | Funding statement |
| **Conflicts of interest** | 28 | Report authors conflicts of interest according to journal or International Committee of Medical Journal Editors requirements. | Competing interests' section |

## Supplementary References


1. White IR, Kalaitzaki E, Thompson SG. Allowing for missing outcome data and incomplete uptake of randomised interventions, with application to an internet-based alcohol trial. *Stat Med* 2011;**30**:3192-207.
2. Jones KC, Weatherly H, Birch S*, et al.* Unit costs of health and social care 2023 manual. https://kar.kent.ac.uk/105685/ Accessed 5th May 2024, 2024.
3. Stevens S, Bankhead C, Mukhtar T*, et al.* Patient-level and practice-level factors associated with consultation duration: A cross-sectional analysis of over one million consultations in english primary care. *BMJ Open* 2017;**7**:e018261.
4. Neilson AR, Bruhn H, Bond CM*, et al.* Pharmacist-led management of chronic pain in primary care: Costs and benefits in a pilot randomised controlled trial. *BMJ Open* 2015;**5**:e006874.
5. NHS Business Services Authority (NHSBSA). Prescription cost analysis, england - 2021/22. https://www.nhsbsa.nhs.uk/statistical-collections/prescription-cost-analysis-england/prescription-cost-analysis-england-202122 Accessed 5th May 2024.
6. British National Formulary (BNF). Last updated 30 april 2025. https://bnf.nice.org.uk/ Accessed 5th May 2025.
7. NHS England. 2022/23 national cost collection data publication. https://www.england.nhs.uk/publication/2022-23-national-cost-collection-data-publication/ Accessed 5th May 2024, 2024.
8. Guy's and St Thomas' NHS Foundation Trust. Appointments: Inflammatory bowel disease (ibd). https://www.guysandstthomas.nhs.uk/our-services/inflammatory-bowel-disease-ibd/appointments Accessed 27th March 2025.
9. National Institute for Health and Care Excellence (NICE). Point-of-care and home faecal calprotectin tests for monitoring treatment response in inflammatory bowel disease. Medtech innovation briefing. 2017. https://www.nice.org.uk/guidance/mib132/resources/pointofcare-and-home-faecal-calprotectin-tests-for-monitoring-treatment-response-in-inflammatory-bowel-disease-pdf-2285963392339141 Accessed 5th May 2024.

10. Akhtar W, Chung Y. Saving the nhs one blood test at a time. *BMJ Open Quality* 2014;**2**:u204012. w1749.
11. Office for National Statistics (ONS) Census 2021. Index of labour costs per hour, uk: July to september 2020 https://www.ons.gov.uk/employmentandlabourmarket/peopleinwork/earningsandworkinghours/bulletins/indexoflabourcostsperhourilch/julytoseptember2020 Accessed 5th May 2024.
12. University of London. Pay and grading - salary scales 01 february 2023. https://www.london.ac.uk/sites/default/files/governance/1-february-2023-interim-pay-award.pdf Accessed 5th May 2024.
13. NHS employers pay scales for 2023/24. Nhs terms and conditions annual, hourly and hcas pay values scales for 2023/24. https://www.nhsemployers.org/articles/pay-scales-202324-archived Accessed 5th May 2024.
