# Supplementary material for: Digital self-management programme for pain, fatigue and faecal incontinence in inflammatory bowel disease: cost-effectiveness analysis of the IBD-BOOST randomised controlled trial
Source: BMJ Open Gastroenterol. 2026 Jul 1;13(1):e002140. doi: 10.1136/bmjgast-2025-002140 (PMC13330898; doi:10.1136/bmjgast-2025-002140)
Supplement: online supplemental file 2 [file bmjgast-13-1-s002.pdf]

Health Economics Analysis Plan (HEAP)

for

Supported online self-management for symptoms of fatigue, pain and urgency/incontinence in people with Inflammatory Bowel Disease

(IBD- BOOST trial)

Version: 1.0
Date: 1st October 2023

Table of Contents

1. ADMINISTRATIVE INFORMATION 5
2. TRIAL INTRODUCTION AND BACKGROUND 7
2.1 Trial background and rational 7
2.2 Aim(s) of the trial 7
2.3 Objectives and/or research hypotheses of the trial 7
2.4 Trial population 8
2.5 Interventions and comparators 8
2.6 Trial design 8
2.7 Trial start and end dates 9
3. ECONOMIC EVALUATION 9
3.1 Aim(s) of the economic evaluation 9
3.2 Objectives of the economic evaluation 9
3.3 Jurisdiction(s) 9
3.4 Perspective(s) 9
3.5 Time horizon(s) 9
4. ECONOMIC DATA COLLECTION & MANAGEMENT 10
4.1 Statistical software 10
4.2 Resources for intervention development 10
4.3 Resources related to online availability and ongoing maintenance of the intervention 11
4.3.1 Resources related to participants' facilitation 12
4.4 Collection of health and other resource data 12
4.5 Costs of resources 15
4.5.1 Intervention costs 15
4.5.2 Healthcare costs 17
4.5.3 Other costs 18
4.6 Analysis of resource use and costs 19
4.7 Health outcomes 19
5. Data analysis 19
5.1 Missing data 19
5.2 Analysis of cost-effectiveness 20
5.2.1 Primary analysis 20
5.2.2 Cost-effectiveness analysis 20
5.3 Subgroup analysis 21
5.4 Sensitivity analyses 21

6. Reporting 22
6.1 Reporting standards 22
6.2 Deviations from the HEAP 22
7. References 23

ABBREVIATIONS

**A&E**	Accident and Emergency
**BNF**	British National Formulary
**CAU**	Care As Usual
**CHEERS**	Consolidated Health Economic Evaluation Reporting Standards
**CEAC**	Cost-Effectiveness Acceptability Curves
**CRF**	Case Report Form
**e-CRF**	electronic Case Report Form
**HEAP**	Health Economics Analysis Plan
**HRQoL**	Health Related Quality of Life
**ICER**	Incremental Cost-Effectiveness Ratio
**NHS**	National Health Service
**NRS**	Numerical Pain Rating Scale
**NHSCII**	NHS Cost Inflation Index
**NICE**	National Institute for Health and Care Excellence
**NIHR**	National Institute for Health and Care Research
**IBD**	Inflammatory Bowel Disease
**IBS**	Irritable Bowel Syndrome
**ITT**	Intention-to-Treat
**MAR**	Missing at Random
**ONS**	Office for National Statistics
**PCA**	Prescription Cost Analysis
**PSS**	Personal Social Services
**QALYs**	Quality-Adjusted Life Years
**QoL**	Quality of Life
**SAP**	Statistical Analysis Plan
**SIV**	Site Initiation Visit

1. ADMINISTRATIVE INFORMATION

**Title**	Health Economics Analysis Plan for the IBD-BOOST trial (a Randomised Controlled Trial of supported online self-management for symptoms of fatigue, pain and urgency/incontinence in people with inflammatory bowel disease)
**Trial registration number**	ISRCTN71618461
**Source of funding**	Funded by the National Institute for Health and Care Research (NIHR); Programme Grant ref number: RP-PG-0216-20001
**Purpose of Health Economics Analysis Plan (HEAP)**	The purpose of this HEAP is to describe the analysis and reporting procedures for the economic analyses planned for the IBD-BOOST trial. The HEAP is designed to ensure that planned health economics analyses are in line with the National Institute for Health and Care Excellence (NICE) technology appraisal recommendations, there is no conflict with the protocol and associated statistical analysis plan and it should be read in conjunction with them.
**Trial protocol version**	This document has been written based on information contained in the trial protocol version 6.0, Date: 21/03/2022
**Trial Statistical Analysis Plan (SAP) version**	SAP Version: 1.0, Date: 18 August 2023
**HEAP revisions**	N/A
**Roles and responsibilities**	The HEAP was prepared by Chris Roukas (health economist) and Borislava Mihaylova (senior health economist) in consultation with Christine Norton (chief investigator) and Tom Hamborg (senior statistician). The trial health economist(s) Chris Roukas and Borislava Mihaylova are responsible for conducting and reporting the economic evaluation in accordance with the HEAP.
**Timing of HEAP in relation to unblinding of data/results**	Version 1.0 of the HEAP was written whilst the health economists had no access to unblinded trial data or to trial results.

Signatures	
**Senior Health Economist**	Signature of senior health economist who is guarantor of the economic evaluation:<br>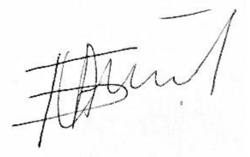<br>Date: 01.10.23
**Senior Statistician**	Signature of senior statistician for the trial:<br>Date: Thomas Hamborg Digitally signed by Thomas Hamborg Date: 2023.10.02 09:23:03 +01'00'
**Chief Investigator**	Signature of chief investigator of the trial:<br>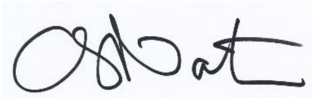<br>Date: 01.10.23

2. TRIAL INTRODUCTION AND BACKGROUND

2.1 Trial background and rational

Inflammatory Bowel Disease (IBD) affects over half a million people (1 in 123) in the UK (Crohn's & Colitis UK, 2022), causing unpredictable bouts of gut inflammation, with acute illness, diarrhoea, and pain. Even when in remission, many people with IBD live with fatigue, chronic abdominal pain, and bowel urgency/incontinence (1). These symptoms of fatigue, pain and urgency/incontinence have a major impact on quality of life in people with IBD but have been largely ignored by clinicians and researchers.

The IBD-BOOST programme, shaped by the concerns of patient and clinician stakeholders, focuses on a supported online self-management intervention for these symptoms. IBD-BOOST is a pragmatic multi-centre two-arm, parallel group superiority randomised controlled trial, with an internal pilot, of Care As Usual (CAU) plus facilitator-supported online self-management versus CAU to manage symptoms of fatigue, pain, and faecal urgency/incontinence in IBD.

2.2 Aim(s) of the trial

IBD-BOOST aims to compare an online delivered self-management programme using the principles of Cognitive Behavioural Therapy with telephone and online messaging support to standard care. The trial will provide evidence for the effectiveness of online delivered self-management programme to improve quality of life for those with IBD-related symptoms of fatigue, pain and urgency.

2.3 Objectives and/or research hypotheses of the trial

The primary objective of the trial is:

1. To establish whether, in people who report symptoms and express a desire for intervention, a facilitator-supported tailored (to patient needs) online self-management programme for fatigue, pain and faecal urgency/incontinence in IBD in addition to CAU improve their Quality of Life (QoL) and global rating of symptom relief 6 months after randomisation, compared with CAU alone.

The secondary objectives of the trial are to determine:

2. If there are any differences between the groups in severity of symptoms of fatigue, pain and urgency/incontinence at 6 and 12 months after randomisation.
3. If there are any differences between the groups in QoL and global rating of symptom relief 12 months after randomisation.
4. If prior medical optimisation of symptoms (Stage 3 of this programme) moderate the treatment response (i.e. do those receiving medical optimisation show greater gains from treatment than those who don't) as measured by the primary outcomes.
5. If people with quiescent IBD at trial commencement have a better response to treatment (primary outcomes) than those with active disease.

6. If baseline depression, or the presence of Irritable Bowel Symptom (IBS), moderate treatment response to intervention.
7. If changes in illness perceptions and behaviours, IBD specific anxiety and self-efficacy, and depression from baseline to 6 months mediate intervention effects on the primary outcomes at 12 months.
8. What are patient’s expectations and experiences of the intervention and what factors may have influenced the intervention implementation.
9. If a facilitator-supported tailored online self-management programme for fatigue, pain and faecal urgency/incontinence in IBD improves generic health-related QoL and is cost-effective.

2.4 Trial population

Inclusion criteria:

- Patients aged 18 years old or over with a diagnosis of IBD (self-reported as having been medically diagnosed with IBD including patients with an ileo-anal pouch or stoma)
- Living in England, Scotland or Wales
- Have participated in Stage 2 of the programme (IBD-BOOST survey) and have self-scored one or more symptoms of fatigue, pain or urgency/incontinence as having an impact on their life of 5 or more on a 0-10 scale when completing Stage 2 (IBD-BOOST survey) or Stage 3 (medical symptom optimisation) – whichever is more recent
- No “red flags” (such as new bleeding, rapid weight loss or vomiting that has not been previously reported to a health care practitioner).
- Access to the online intervention via a computer or mobile device

Exclusion criteria:

- One or more “red flags”
- Inability to give informed consent (for example, due to reduced mental capacity)
- Insufficient command of English to understand study documents and procedures

2.5 Interventions and comparators

Intervention: Access to CAU plus the BOOST online tailored self-management programme for 6 months, plus one individual telephone or Skype support session (for up to 30 minutes, training and a paper copy of the content will be provided for the facilitator), plus access to online messaging with their facilitator via the BOOST platform for the first 3 months after recruitment.

Comparator: Patients in the CAU group will have access to all usual care, including monitoring with clinic visits and/or via the local IBD helpline.

2.6 Trial design

A pragmatic multi-centre two-arm, parallel group superiority randomised controlled trial, with an internal pilot, of facilitator-supported online self-management versus CAU

to manage symptoms of fatigue, pain, and faecal urgency/incontinence in IBD. One baseline assessment and two assessments at 6 and 12 months after randomisation will be carried out.

2.7 Trial start and end dates

Recruitment started in February 2019 and was completed in July 2022. The 12-month follow-up period was completed in March 2023.

3. ECONOMIC EVALUATION

3.1 Aim(s) of the economic evaluation

An economic evaluation will be conducted as part of the trial. The aim of the economic evaluation is to address the question "***in people with IBD, what is the cost-effectiveness of CAU plus BOOST online tailored self-management programme compared with CAU alone***".

The within-trial economic analysis will be performed using the individual patient level data collected in the IBD-BOOST trial. The analytical approach will take the form of a cost-utility analysis. Incremental cost-utility ratios will be calculated by taking a ratio of difference in the mean costs and mean quality-adjusted life years (QALYs) between treatment arms.

3.2 Objectives of the economic evaluation

The primary objective of the economic evaluation is:

1. To estimate in people with IBD the cost-effectiveness of CAU plus BOOST online tailored self-management programme compared with CAU alone at 12 months post randomisation.

3.3 Jurisdiction(s)

The trial is conducted in the UK which has a National Health Service (NHS), providing publicly funded healthcare, primarily free of charge at the point of use.

3.4 Perspective(s)

The economic analysis will be undertaken from the UK NHS and personal social services (PSS) and, separately, from the societal perspectives.

3.5 Time horizon(s)

The economic analysis will compare the costs and QALYs of each group over the 12 months after randomisation.

4. ECONOMIC DATA COLLECTION & MANAGEMENT

4.1 Statistical software

All analysis will be carried out using STATA software. The relevant STATA version number will be recorded in the health economics report.

4.2 Resources for intervention development

Similar to other online health technologies (2), the IBD-BOOST intervention development included four categories of resources (**Table 1**):

1. Development of the 11 online sessions: Sessions 1-7, are core transdiagnostic sessions to be completed by participants experiencing fatigue, pain and faecal urgency/incontinence and sessions 8-11 are symptom-specific sessions to be completed by participants experiencing or with a specific interest in fatigue, pain and faecal urgency/incontinence, respectively. These sessions were developed with extensive patient and clinician engagement;
2. Development of the web-application as a platform to deliver the IBD-BOOST intervention (consisting of reporting a database specification to support the development of the web-application, developing source code for the web-application (software development), conceptualising and developing a recommender system into the web-application codebase, and designing the intervention to ensure it was appealing to both operate aesthetically (graphic design) and practically (interaction design);
3. Additional communications necessary to develop the intervention consisting of IBD-BOOST study team task meetings for general discussions, steering committee meetings to advice on safety and ethical concerns and intervention development group meetings to provide feedback on intervention;
4. Testing the intervention consisting of testing the intervention's performance on the web-application including the collection of outcome data and user feasibility testing prior to the pilot study.

Table 1: Summary of IBD-BOOST intervention development resource use categories

**Resource use categories for the development of intervention**
Development of IBD-BOOST sessions<br>• Staff time to develop IBD-BOOST sessions<br>• Staff time to secure ethical approval<br>• Steering Group meetings
Web application development<br>• Database specifications<br>• Software development<br>• Recommended system and integrating into the codebase<br>• Graphic design<br>• Interaction design

Communication<br>• Task meetings<br>• Advisory Board meetings<br>• Design group meetings
Intervention<br>• Intervention testing<br>• Feasibility testing

Most of the development costs, excluding intervention implementation into web application, will not be taken into account in our analysis. These costs are related to research funding rather than NHS and PSS resources and unlikely to be repeated if the intervention is adopted in practice.

4.3 Resources related to online availability and ongoing maintenance of the intervention

The intervention consists of an online tailored self-management programme (11 sessions) for 6 months. In the study the BOOST intervention was internally provided as part of the research programme. In practice, if implemented, it would need to be integrated into the NHS practice. The resources required for the web implementation and ongoing maintenance of the intervention (Table 2) will based on an earlier study of an online intervention for diabetes management (3).

Table 2: Resources of ongoing maintenance and delivery of BOOST

	**Activities**	**Intensity**
Hardware and software	Functional and technical specifications (inc. domain purchase)	12 months
	Set-up	12 months
	Website maintenance	12 months
	Project management	12 months
Further Staff involvement in [illegible]	Content checking, revising and updating by research staff	1 hour each 2 weeks

	Content checking, revising and updating by clinical lead	1hour/2 weeks

4.3.1 Resources related to participants' facilitation

Participants in the intervention group were provided with facilitation by 16 research nurses to encourage use of the BOOST intervention. Research nurses required training in providing the intervention. Each nurse attended both face-to-face and group training sessions provided by a member of the research team. Research nurses were also provided with a paper copy of the content of the BOOST. The intervention included assess to the online intervention plus one individual telephone or Skype support session (for up to 30 minutes), plus access to online messaging with their facilitator via the BOOST platform for the first 3 months after recruitment. Staff time for the initial support sessions with patients and for the messaging contacts in the first 3 months will be included. Individual and group supervision sessions were also provided to all participating facilitators' of the study to ensure the consistent and, more importantly, safe delivery of BOOST facilitation.

4.4 Collection of health and other resource data

All participants will have access to all usual care, including monitoring with clinic visits and/or via the local IBD helpline.

Use of primary and secondary care services and other resource will be collected over the 12-month follow-up period via electronic Case Report Form (e-CRF) using a study-specific self-reported IBD Resource Use questionnaire developed within this Programme Grant. The questionnaire will capture healthcare (NHS) resource use, costs borne by the patient related to their IBD and lost productivity costs (**Table 3**).

The analysis from the NHS and PSS perspective will include the healthcare and social care used measured by the frequency of use of inpatient care, outpatient care, diagnostic procedures, medications and any social care.

In addition, the analysis from the societal perspective will also include (1) the out-of-pocket expenses, measured by participants' personal expenses on medications, products and complementary therapies, and travel to and from IBD appointments, care from family and friends, and (2) productivity losses, measured using information about participants' employment status and days off-work due to IBD in the previous three months collected in the questionnaire.

The IBD Resource Use questionnaire will be completed by the participant covering three periods (3 months pre-randomisation to baseline/randomisation, 3 months to 6 months post-randomisation and 9 months to 12 months post-randomisation).

Table 3: Summary of resource use in the IBD-BOOST trial

**Resource use item**	
**BOOST intervention**	
• Maintenance and updating of BOOST platform	Invoices / Staff grade
• Facilitators' 1-2-1 and group supervision	Researcher case report form (CRF): Time spent on supervision
➢ Printed materials	Invoice
• Delivery of BOOST intervention to patients, including:	From BOOST platform: Facilitator time / Staff grade
➢ Messages sent to patients via BOOST platform	Number of messages
➢ Text sent to patients	Number of texts
➢ Number of phone/Skype calls to patients	Time spent on phone
• Facilitator's training (includes initial training, follow-up training, practice with patient, Site Initiation Visit (SIV) training and top-up training)	Researcher CRFs: Time spent on training / Travel time and other cost
**Healthcare**	**Source: e-CRF (recall period: 3 months)**
**Outpatient visits**	
❖ **Specialist doctor**	
• Gastroenterologist	Number of visits
• Colorectal surgeon	Number of visits
• Radiologist	Number of visits
• Rheumatologist	Number of visits
• Other specialist doctor	Number of visits
❖ **Other health professional**	
• IBD nurse	Number of visits
• IBD advice line	Number of visits
• Stoma nurse	Number of visits
• Accident and Emergency (A&E) staff	Number of visits
• General Practitioner	Number of visits
• General practice nurse	Number of visits
• Dietician	Number of visits
• Psychologist	Number of visits
• Pharmacist	Number of visits
• Other health professional	Number of visits
**Diagnostic procedures**	
• CT scan	Number of tests
• MRI scan	Number of tests
• Colonoscopy	Number of tests
• Upper GI Endoscopy	Number of tests

• Ultrasound	Number of tests
• Stool test (e.g. faecal calprotectin test)	Number of tests
• Blood test	Number of tests
• Any other diagnostic test	Number of tests
**Medication**	
• Aminosalicylates / 5ASA by mouth	Use Yes/No
• Aminosalicylates / 5ASA as an enema or suppository	Use Yes/No
• Azathioprine or mercaptopurine	Use Yes/No
• Steroids as an enema or suppository (e.g.	Use Yes/No
• Methotrexate by mouth or as an injection	Use Yes/No
• Infliximab as an infusion	Use Yes/No
• Vedolizumab as an infusion	Use Yes/No
• Golimumab as an injection	Use Yes/No
• Adalimumab as an injection or infusion	Use Yes/No
• Ustekinumab as an injection or infusion	Use Yes/No
• Other medication	Use Yes/No
• Steroids by mouth	Use Yes/No
**Hospital admissions: f**or each hospital admission	
• Number of nights spent in hospital	
• Number of days in intensive care	
• Type of operation	
**Care from social services/other public services**	**Source: e-CRF (recall period: 3 months)**
• Number of weeks and weekly hours of help	
**Out-of-pocket expenses**	**Source: e-CRF (recall period: 3 months)**
❖ **Symptom management**	
• Anti-diarrhoeal medication	Cost (£)
• Pain killers	Cost (£)
• Iron supplements	Cost (£)
• Vitamin supplements	Cost (£)
• Rehydration solutions	Cost (£)
• Meal replacement	Cost (£)
• Lifestyle	Cost (£)
❖ **Products**	
• Pads and/or pants for faecal incontinence	Cost (£)
• Wet wipes	Cost (£)
• Air fresheners	Cost (£)
• Creams	Cost (£)
• Bed protection	Cost (£)
❖ **Complementary & Alternative Therapies**	
• Herbal supplements	Cost (£)
• Probiotics	Cost (£)

• Prebiotics	Cost (£)
• Fish oil	Cost (£)
• Acupuncture/Massage/Relaxation	Cost (£)
• Psychological therapy	Cost (£)
• Other therapies	Cost (£)
❖ **Travel**	
• By car	Cost (£)
• Bus/tube/train	Cost (£)
• Taxi	Cost (£)
• Other	Cost (£)
**Care from family or friends**	**Source: e-CRF (recall period: 3 months)**
• Number of weeks and weekly hours of help	
**Employment**	**Source: e-CRF (recall period: 3 months)**
• Number of days off work due to IBD	

To improve completion rate of the questionnaires, two email or text reminders will be sent for those who do not respond to the initial questionnaire as well as to those not responding to the 6-month and 12-month follow-up questionnaires.

4.5 Costs of resources

Costs for 2022-23 year will be used throughout the assessment. Costs will not be discounted as the duration of follow-up in the study is 12 months.

4.5.1 Intervention costs

There were two types of costs related to the intervention: those incurred during the development and optimisation of the intervention and those related to web implementation of the intervention and ongoing delivery and maintenance of the intervention.

The costs relating to web implementation and ongoing maintenance and delivery of the intervention within the trial inform intervention delivery cost in base case analysis. These consist of the cost of web implementation, delivery of the intervention, the cost of maintenance and updating of the intervention and the cost of facilitating activities undertaken to improve uptake and use. If the BOOST platform was to be widely implemented into routine healthcare, all these activities would be required on an on-going basis. Therefore, the costs of these activities that occurred during the trial will be used to estimate the real costs in practice.

Costs related to hardware, software and work undertaken by the third-party service provider were recorded from actual invoices. Costs related to activities undertaken by research or academic staff will be estimated from workloads during the trial period, by recording the time taken for each activity, the frequency of that activity and the number and grade of staff involved. The facilitator time will be costed at NHS grade of role

providing the service if BOOST intervention implemented in NHS. These included initial introductory calls and interaction with patients on the website (messages, Skype calls). The costs will be calculated by multiplying the time spent by the average wage for each type of staff member. Hourly costs of NHS staff will be taken from unit costs of health and social care edited by Personal Social Services Unit (PSSRU) (8)

Research nurses acting as BOOST facilitators also required training in providing the BOOST intervention to participants. All facilitators attended training on the delivery of the intervention followed by a practice patient, SIV training and top-up training if necessary. The costs of the training will be calculated from the time spent on each activity by the nurses and research team staff, plus travel time for the nurses multiplied by their respective hourly rates. Supervision provided by research team staff will also be estimated by the time spent on supervision multiplied by respective hourly rates assuming training will be provided by NHS staff (**Table 4**). All intervention costs will be allocated to the participants in the intervention group of the trial to give a per participant cost.

Table 4: BOOST intervention cost used in the analysis

**BOOST component**	**Intensity**	**Unit cost (£)**	**Sources**
Functional and technical specifications (inc. domain purchase)	12-month period	30,000	Third-party invoice
Set-up	12-month period		Third-party invoice
Website maintenance	12-month period		Third-party invoice
Project management	12-month period		Third-party invoice
Content checking, revising and updating by staff	1hour/2 weeks	17	Grade 6 staff
Content checking, revising and updating by clinical team	1hour/2 weeks	29	Academic research and teaching Level 8, scale 41
Facilitators trainers	Total hours of supervision	17 (NHS Band 6)	NHS Grade
Facilitators training	Total hours of training / Travel time in hours / Travel expenses	17 (Band 6)<br>22 (Band 7)	NHS Grade

Printing materials (for facilitator)	1 copy per facilitator for the 12-month period		

4.5.2 Healthcare costs

Data on health care resource use were collected retrospectively from participants using a self-report questionnaire online. These data were collected at baseline for the 3-month period before the trial, at 6-month follow-up for months 4-6 after randomisation and at 12-month follow-up from months 9-12 after randomisation. The quantities will then be multiplied by a set of national average unit costs to derive costs of resources (**Table 5**).

Table 5: National average unit cost used in the analysis

Health Service Use	Unit cost (£)	Sources
**Specialist doctor**		
Gastroenterologist	183	2021/22 National Schedule of NHS costs (4)
Colorectal surgeon	146	2021/22 National Schedule of NHS costs
Radiologist	85	2021/22 National Schedule of NHS costs
Rheumatologist	188	2021/22 National Schedule of NHS costs
**Other health professional**		
IBD nurse	53	PSSRU 2022 (cost per working hour) (5)
IBD advice line / helpline	9	PSSRU 2022 (nurse led)
Stoma nurse	53	PSSRU 2022 (cost per working hour)
Accident and emergency service	166	2021/22 National Schedule of NHS costs
GP	41	PSSRU 2022
Practice nurse	13	PSSRU 2022 (15min consultation in surgery)
Dietician	98	2021/22 National Schedule of NHS costs (non-consultant led)
Psychologist	208	2021/22 National Schedule of NHS costs (non-consultant led)
Pharmacist	17	Csikar JI et al, 2016 (20min appt) (6)
**Diagnostic procedures**		

CT scan	114	2021/22 National Schedule of NHS costs (weighted average of all adult Attendances)
MRI scan	276	2021/22 National Schedule of NHS costs (weighted average of all adult Attendances)
Colonoscopy	233	2021/22 National Schedule of NHS costs (weighted average of all adult Attendances)
Upper GI endoscopy	194	2021/22 National Schedule of NHS costs (weighted average of all adult Attendances)
Ultrasound	104	2021/22 National Schedule of NHS costs (weighted average of all adult Attendances)
Stool test	30	NICE Faecal calprotectin diagnostic tests for inflammatory diseases of the bowel (7)
Blood test	11	includes FBC,LFT,U&E,CRP (8)
IBD admission (per night)	514	2021/22 National Schedule of NHS costs (weighted average of all adult Attendances)

Costs of Prescribed items will be informed from the Prescription Cost Analysis (PCA) England 2022-23 (10) using their generic name and form where available. As the medication dose range and quantities are not collected, we will assume a mean cost for each medication item based on the PCA, which derives products' net ingredient cost (i.e. excluding discounts and dispensing costs) using the corresponding average duration of prescription. Unless it was specified that medication was prescribed, blank entries will be considered as not used.

4.5.3 Other costs

Collection of costs for direct nonmedical resource items such as over the counter medication, products and supplementary services, help with childcare and travel to appointments incurred by the participant will be obtained directly from the e-CRF. Productivity loss will be estimated by multiplying the self-reported number of days off-work due to IBD by the daily/annual wage provided by the Office for National Statistics (ONS) (10).

4.6 Analysis of resource use and costs

The health economics analysis will be undertaken from the perspective of the NHS and PSS following the National Institute of Health and Care Excellence (NICE) guidance (12) and, separately, from societal perspective. The analysis will follow an intention-to-treat strategy with the patients allocated to BOOST intervention incurring the cost of treatment.

All costs will be reported in British pound sterling (£) at 2022-2023 prices. Where necessary costs will be adjusted for inflation using the NHS Cost Inflation Index (NHSCII) (5).

4.7 Health outcomes

The participants' questionnaires contain the EQ-5D-5L for self-completion at baseline, 6 months and 12 months post randomisation. The EQ-5D-5L instrument (13) facilitates the generation of a utility score from a person's health related quality of life while reducing the ceiling effect and being more sensitive than its three-level (3L) predecessor (14). As per the NICE's latest guide on health technology evaluations (15) the EQ-5D-5L utility values will be calculated by mapping into the UK EQ-5D-3L value using the mapping function developed by the Decision Support Unit using the 'EEPRU dataset' (16). Quality-adjusted life years (QALYs) will then be calculated for each participant over the first 6 months of follow-up and, separately, the second 6 months, using the area under the curve of utility values from the three time points. QALYs will not be discounted because the follow-up period is 12 months.

5. Data analysis

5.1 Missing data

Incomplete data are a particular issue in within-trial health economic evaluations and can result from time-point missingness. We will consider data as missing if a participant provided a partially completed questionnaire or was lost to follow-up. Missing data of continuous variables of interest (cost / resource use by category, QoL utilities) at baseline will be imputed using mean imputation. Individual questions without responses on both resource use and number of visits or contacts in the returned questionnaires will be assumed to indicate that no such resource use had taken place over the past three months. The remaining missing data will be imputed using multiple imputation with chained equations and predictive mean matching with number of donors (knn in Stata) equal to 10 (17). This approach assumes that data are missing at random (MAR), i.e. that the value of the missing data on costs and/or Health Related Quality of Life (HRQoL) could be predicted from the non-missing data.

As per SAP, the number of the imputed dataset will mirror the proportion of participants with at least one missing value (for example, if 25% of study participants have at least one missing value, we will then use 25 imputed datasets for our analysis). If there is a

very high percentage of incomplete cases, the average percentage of incomplete cases will be used as the number of imputations required.

We will finally estimate overall means and standard errors for components of cost-effectiveness by combining the estimates obtained from each imputed dataset using Rubin's rules (17).

5.2 Analysis of cost-effectiveness

5.2.1 Primary analysis

A cost-utility analysis will be conducted from a Health and Personal Social care and separately, a societal perspective using the IBD-BOOST trial data over a 12-month time horizon.

The following outcomes, measured over the first 6 months and subsequent 6 months of follow up will be compared: (1) QALYs; (2) total costs from NHS and PSC perspective; (3) Other costs included in the societal perspective. The comparisons will employ the statistical model used for the analysis of the primary outcome in IBD-BOOST (see SAP). Namely, a partially nested mixed-effects model will be employed. The clustering effect (of patients nested within intervention facilitators) will be modelled only in the intervention arm. Participants in the control arm will be treated as independent. In the intervention only, a random slope will be specified to allow the effect of treatment to vary between intervention facilitators. An unstructured covariance matrix will be used for the residual errors of repeated measures over time. An interaction effect for randomised treatment group and post-randomisation time point (as a categorical variable) will be fitted to achieve a saturated model that allows estimation of mean estimates at each time point in each treatment group.

The covariates specified for the analysis of the primary outcome in IBD-BOOST will be employed: baseline value of the outcome (with baseline EQ-5D utility for QALYs outcome), stratification factors; PROMIS fatigue, PROMIS pain, and PROMIS incontinence at baseline as continuous covariates, participant age (continuous variable), and participant gender (categorical variable).

If any model fails to converge, we will use the strategy specified in SAP of removing covariates and design effects.

5.2.2 Cost-effectiveness analysis

The analysis will adopt intention-to-treat (ITT) principles. The base-case analysis will rely on costs and outcomes estimated for the whole trial population.

The comparisons described in section 5.2.1 will be used to derive overall QALYs over the 12 months follow-up in the trial and overall costs from the NHS and PSS, and separately, from societal perspective. As the trial collected information over 3 months follow-up in the first 6 months of follow-up and 3 month in the second 6 months, the 12-monthly overall cost differences will be estimated as two times the estimated cost differences in respective follow-up periods.

The NICE (18) cost-effectiveness thresholds of £20,000 to £30,000 per additional QALY will be used to identify whether the intervention represents good value for money. Measures of uncertainty (standard errors and confidence intervals) will also be reported around the mean costs and QALYs and confidence intervals will be presented. Probability of cost-effectiveness will be reported across values of the cost-effectiveness threshold.

5.3 Subgroup analysis

The SAP included a pre-specified subgroup analysis to examine differences in outcome defined by baseline characteristics. These baseline characteristics are:

- IBD in remission or not: IBD remission at baseline is defined as faecal calprotectin <200μg/g AND an IBD control score ≥13.
- PHQ-9 measure of depression at baseline: not depressed or depressed. Not depressed at baseline is defined as PHQ-9 = 0-9 and depressed at baseline is defined as PHQ-9 score = 10-27.
- Visceral Sensitivity Index (VSI) measures gastrointestinal, symptom-specific anxiety using 15 questionnaire items, with responses ranging from 1 (i.e., strongly agree) to 6 (i.e., strongly disagree). Higher scores indicate more severe, symptom-specific anxiety. This subgroup analysis will investigate whether trial participants with symptom-specific anxiety at baseline experience a different response to the individually tailored, online, self-management intervention compared to trial participants who do not have symptom-specific anxiety.
- Rome IV criteria for IBS met at baseline or not. The Rome IV criteria are used for the diagnosis of IBS. IBS is present when specific items (listed in SAP) from the RSD are scored as 1:

We will perform subgroup analyses by including an interaction term, one at a time, between treatment arm and each of the baseline characteristics above testing for the significance of the interaction effect.

5.4 Sensitivity analyses

As the cost for the web implementation and ongoing maintenance of the intervention can vary between NHS Trusts (i.e. the whole programme could be one part of a whole-sale deal of IT services or they could outsource it to a private company), we will present sensitivity analysis for results varying this cost from £5K to £50K.

In the base-case analysis, we estimated the supervision cost by recording the trial member (offering supervision) research time. However, in clinical practice, the cost for clinical supervision is estimated 30% higher by the clinical team. As a sensitivity

analysis, we will therefore perform an analysis by adjusting supervision costs to NHS practice and evaluate whether the intervention is cost-effective.

In the base-case analysis we impute missing data also when entire questionnaires are missing. In a sensitivity analysis, we will report cost-effectiveness results without imputing data in cases where entire follow-up questionnaires were not returned.

6. Reporting

6.1 Reporting standards

Findings of this economic evaluation will be reported in accordance with the Consolidated Health Economic Evaluation Reporting Standards (CHEERS) (19) statement for the reporting of health economic evaluations.

6.2 Deviations from the HEAP

Any deviation from HEAP will be described and justified in the final report.

7. References

1. Wilson BS, Lonnfors S, Vermeire S, Greco M, Hommes DW, Bell C, et al. The true impact of IBD: a European Crohn's and Ulcerative Colitis patient life impact survey 2010-11. 2012 2012.
2. Paterson, L., Rennick-Egglestone, S., Gavan, S.P., Slade, M., Ng, F., Llewellyn-Beardsley, J., Bond, C., Grundy, A., Nicholson, J., Quadri, D. and Bailey, S., 2022. Development and delivery cost of digital health technologies for mental health: Application to the Narrative Experiences Online Intervention. Frontiers in Psychiatry, 13, p.1028156.
3. Li, J., Parrott, S., Sweeting, M., Farmer, A., Ross, J., Dack, C., Pal, K., Yardley, L., Barnard, M., Hudda, M. and Alkhaldi, G., 2018. Cost-effectiveness of facilitated access to a self-management website, compared to usual care, for patients with type 2 diabetes (HeLP-Diabetes): randomized controlled trial. Journal of medical Internet research, 20(6), p.e201.
4. NHS England, 2021/22 National Cost Collection Data Publication 2022. https://www.england.nhs.uk/costing-in-the-nhs/national-cost-collection/. Accessed on 14 September 2023
5. Jones, Karen C. and Weatherly, Helen and Birch, Sarah and Castelli, Adriana and Chalkley, Martin and Dargan, Alan and Forder, Julien E. and Gao, Jinbao and Hinde, Seb and Markham, Sarah and Ogunleye, Della and Premji, Shainur and Roland, Daniel (2023) Unit Costs of Health and Social Care 2022 Manual. Technical report. Personal Social Services Research Unit (University of Kent) & Centre for Health Economics (University of York), Kent, UK 10.22024/UniKent/01.02.100519. (doi:10.22024/UniKent/01.02.100519)
6. Csikar, J.I., Douglas, G.V., Pavitt, S. and Hulme, C., 2016. The cost-effectiveness of smoking cessation services provided by general dental practice, general medical practice, pharmacy and NHS Stop Smoking Services in the North of England. Community Dentistry and Oral Epidemiology, 44(2), pp.119-127.
7. National Institute for Clinical Excellence, 2013. Faecal calprotectin diagnostic tests for inflammatory diseases of the bowel. Diagnostics guidance DG11. National Institute for Clinical Excellence. Available: https://www. nice. org. uk/guidance/dg11 [Accessed 14 September 2023].
8. Akhtar, W. and Chung, Y., 2014. Saving the NHS one blood test at a time. BMJ Open Quality, 2(2), pp.u204012-w1749.

Prescription Cost Analysis – England – 2021/22 | NHSBSA [Internet]. Available from: https://www.nhsbsa.nhs.uk/statistical-collections/prescription-cost-analysis-england/prescription-cost-analysis-england-202122. Accessed on 01 August 2023.

9. Jones, Karen C. and Weatherly, Helen and Birch, Sarah and Castelli, Adriana and Chalkley, Martin and Dargan, Alan and Forder, Julien E. and Gao, Jinbao and Hinde, Seb and Markham, Sarah and Ogunleye, Della and Premji, Shainur and Roland, Daniel (2023) Unit Costs of Health and Social Care 2022 Manual. Technical report. Personal Social Services Research Unit (University of Kent) & Centre for Health Economics (University of York), Kent, UK 10.22024/UniKent/01.02.100519. (doi:10.22024/UniKent/01.02.100519) (KAR id:100519)
10. Drug Tariff | NHSBSA [Internet]. Available from: https://www.nhsbsa.nhs.uk/pharmacies-gp-practices-and-appliance-contractors/drug-tariff. Accessed on 01 August 2023.

11. Office for National Statistics. Earnings and hours worked, age group: ASHE Table 6. In:2022. Available from: https://www.ons.gov.uk/employmentandlabourmarket/peopleinwork/earningsandworkinghours/datasets/agegroupashetable6. Accessed on 01 August 2023.
12. National Institute for Clinical Excellence, 2008. Guide to the methods of technology appraisal. https://www.nice.org.uk/process/pmg9/chapter/foreword
13. Herdman, M., et al., Development and preliminary testing of the new five-level version of EQ-5D (EQ-5D-5L). Quality of life research, 2011. 20(10): p. 1727-1736
14. Rabin R, Oemar M, Oppe M, Janssen B, Herdman M. EQ-5D-3L user guide. Basic information on how to use the EQ-5D-5L instrument. Rotterdam: EuroQol Group. 2011;22.
15. National Institute for Health and Care Excellence (NICE). (2022, January 31). NICE health technology evaluations: the manual. Retrieved 06 August, 2022, from https://www.nice.org.uk/process/pmg36/chapter/introduction-to-health- technology-evaluation
16. Hernández Alava, M., Pudney, S. and Wailoo, A., 2023. Estimating the relationship between EQ-5D-5L and EQ-5D-3L: results from a UK population study. PharmacoEconomics, 41(2), pp.199-207.White, I.R., Royston, P. and Wood, A.M., 2011. Multiple imputation using chained equations: issues and guidance for practice. Statistics in medicine, 30(4), pp.377-399.
17. Rubin DB. Multiple Imputation for Nonresponse in Surveys. Wiley: New York, 1987.
18. National Institute for Health and Care Excellence, 2013. Judging whether public health interventions offer value for money. Local Government Briefing (LGB)
19. Husereau D, Drummond M, Augustovski F, de Bekker-Grob E, Briggs AH, Carswell C, et al. Consolidated Health Economic Evaluation Reporting Standards 2022 (CHEERS 2022) Statement: Updated Reporting Guidance for Health Economic Evaluations. Value in Health. 2022;25(1):3–9.
